# Bridged Azobenzene Exhibits Fully Reversible Photocontrolled Binding to a G‑Quadruplex DNA/Duplex Junction

**DOI:** 10.1021/jacsau.5c00532

**Published:** 2025-08-07

**Authors:** Javier Ramos-Soriano, Y. Jennifer Jiang, Bowen Deng, Michael P. O′Hagan, Aditya G. Rao, Doudou Lu, Susanta Haldar, A. Sofia F. Oliveira, Adrian J. Mulholland, M. Carmen Galan

**Affiliations:** † School of Chemistry, 152332 University of Bristol, Cantock’s Close, Bristol BS8 1TS, United Kingdom; ‡ Centre for Computational Chemistry, 1980 University of Bristol, Cantock’s Close, Bristol BS8 1TS, United Kingdom

**Keywords:** DNA nanodevices, photoswitch, azobenzene, G4/duplex DNA junction, supramolecular DNA interactions

## Abstract

The ability to selectively control DNA conformation using light as an external stimulus offers unique opportunities to control specific DNA sequences in biological settings and to develop nucleotide-based nanodevices. We describe a duplex/G-quadruplex (G4) junction-binding chemotype derived from a cyclic azobenzene core that reversibly photoswitches between *cis* and *trans* isomers, mediated exclusively by visible light under physiological conditions. We demonstrate the selective binding of the elongated *trans* conformation, with over 50-fold higher affinity, toward LTR-III G4 (an important HIV-1 sequence), and show that binding and dissociation from the LTR-III G4 can be controlled reversibly by alternate irradiation with low-intensity blue and green light. NMR and MD simulations indicate that the different isomers exhibit very distinct binding modes. While the elongated *trans* ligand preferentially binds at the G4/duplex junction of the LTR-III sequence, a DNA motif which is gaining increasing attention as a potential drug target, the bent *cis* isomer favors the duplex region.

## Graphical abstract

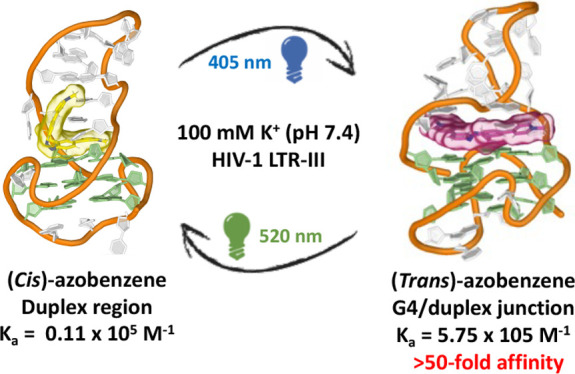

## 1 Introduction

DNA is a highly dynamic biomolecule, and it is well known that its conformational polymorphism plays a key role in biological systems.^1^ For example, many proteins bind to A-form DNA,^2^ while supercoiled (mechanically stressed) DNA plays an important role in transcription.^3^ The ability to reversibly induce, trap, or drug such transient DNA conformations with high spatiotemporal precision using stimuli-responsive ligands would open up new frontiers in molecular biology, pharmacology, and systems chemistry.^4^ However, achieving the robust, selective, and reversible binding of noncanonical DNA structures by small-molecule ligands remains an elusive goal.

Among the diversity of noncanonical DNA structures, G-quadruplexes (G4s) represent a high priority target, owing to mounting evidence for their prevalence in mammalian genomes, as well as in those of other organisms, including plants, bacteria, and viruses.^5^ Briefly, G4s are comprised of stacked arrangements of G-tetrads formed by the self-association of four guanine residues into a square-planar arrangement stabilized by Hoogsteen hydrogen bonding and coordination to a central metal cation.^6^ The transient formation of G4s in vivo has been linked to essential genomic functions, such as transcription, replication, repair, and telomere maintenance,^5,7–10^ and the selective targeting of G4s with small-molecule ligands has revealed a variety of promising therapeutic effects, both in cellular models and in whole organisms.^11,12^

A limitation of many reported G4 ligands is their G4 binding promiscuity, as they are generally derived from planar aromatic scaffolds that target common features of G4s (G-tetrads) rather than features unique to specific G4s, such as loops and grooves, which pose a greater ligand design challenge. Moreover, G4 ligand design and screening studies generally investigate ligand binding to G4 in isolation, thus overlooking the effects of flanking or embedded duplex sequences that may influence ligand binding in the genomic environment.^13^

In addition, the static nature of the majority of G4 ligands greatly limits the extent to which their activity can be controlled in a dynamic system. Recent examples from our research group^14,15^ have been focused on addressing this challenge by engineering light-responsive ligands for which G4 binding activity can be toggled in a noninvasive and controlled manner through photoisomerization of the ligand scaffold.^14^ Moreover, pioneering examples of light-activated G4-targeting photocages,^16^ photoconvertible ligands,^17^ photosynthesizers,^18^ and photoswitches^19–21^ have demonstrated the potential of these types of ligands to photocontrol G4-folding dynamics, cytotoxicity, and gene transcription.^22^

Despite significant progress, all of the reported systems suffer from limitations that preclude real-world applications, including reliance on short-wavelength light (low tissue penetration), loss of efficacy in physiological conditions (for example, high K^+^ concentration),^23,24^ insufficient differences in G4 affinity/activity between the photoisomers,^25^ or photofatigue.^26,27^ The robust photocontrol of physiological G4-based systems remains an elusive and challenging goal.

In this study, we addressed the above limitation by focusing our attention on a novel class of photoresponsive ligands based on a cyclic-bridged azobenzene chromophore with a unique molecular geometry and superior photoswitching properties. As the DNA model, we selected a distinctive G4 in the U3 promoter region of the HIV-1 long terminal repeat (LTR-III) comprised of a three-layer (3 + 1) G4 scaffold and a 12-nt duplex diagonal loop forming a G4/duplex junction (Figure 1a), identified by Phan and co-workers.^28^ The team proposed this unique quadruplex–duplex junction, which combines a highly dynamic duplex base pair at the boundary between the duplex and G4 moieties, as a potential druggable target. In fact, the targeting of these junctions has been proposed as a promising strategy to overcome affinity/selectivity issues between G4s and duplex DNA structures.^13,29^ Pioneering works have employed hybrid ligands that could simultaneously interact with the G4 and duplex minor groove in a given sequence.^13,30^ Additionally, the known G4-specific ligands (i.e., aminomethyl-substituted aromatic compounds), such as indoloquinoline,^31,32^ naphthalene diimide,^33^ pyrodostatin,^34^ and camptothecin^35^ derivatives, DOXO,^35^ BRACO-19,^36^ among others,^37^ have been used as G4/duplex junction binders. However, although G4/duplex junctions are frequent in genomic sequences,^38–40^ the design of ligands targeting specifically the junction is still a challenge.

**Figure 1:**
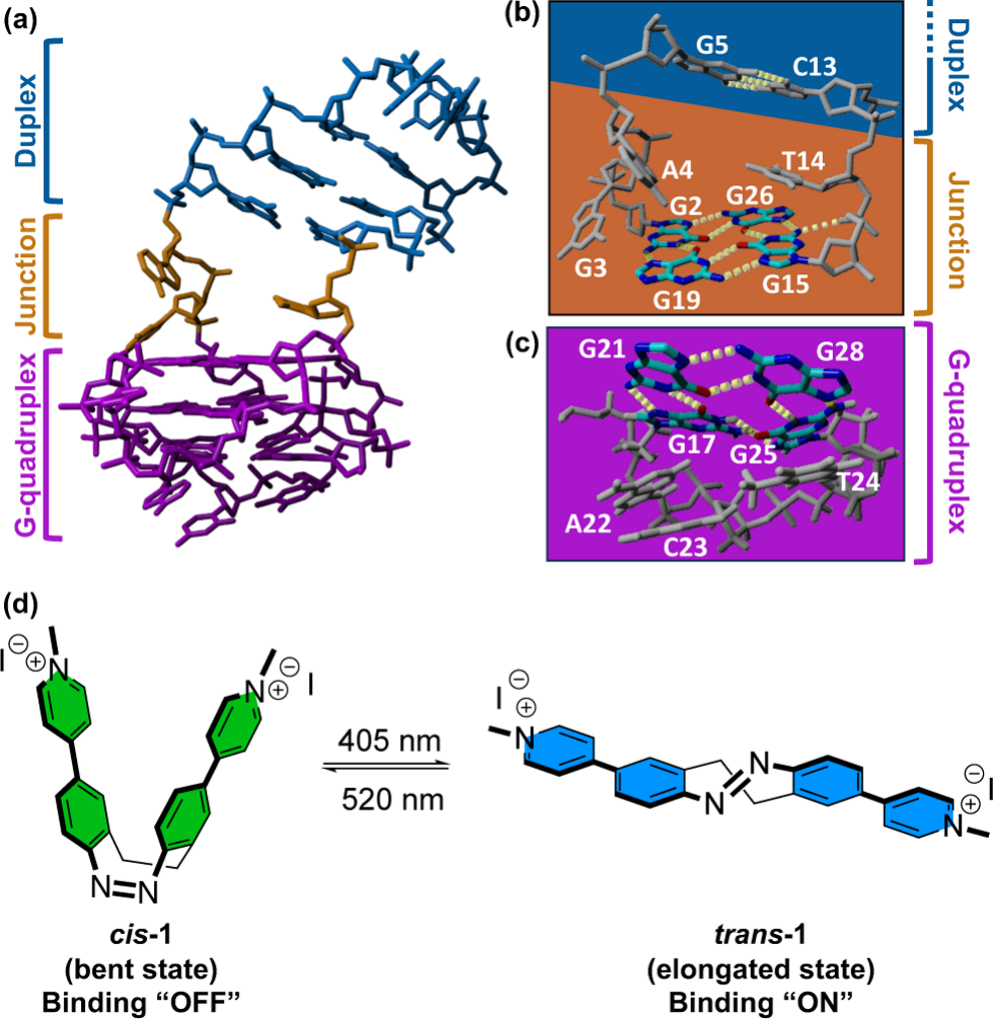
Targeting unique structural features of the LTR-III G4/duplex hybrid with a photoresponsive ligand. (a) NMR structure (PDB: 6H1K, pose 1) of the full sequence showing the G4 (purple), junction (orange), and duplex (blue) domains, (b, c) zoom-in representation of potential ligand binding sites on the (b) top G4 (G2, G15, G19, G26) flanked by the A4, G4, C13, T14 duplex junction (gray), and (c) bottom G4 (G17, G21, G25, G28) flanked by the A22, C23, T24 lateral loop (gray). (d) Novel photoisomerizable-bridged azobenzene ligand **1** reported in the present study.

Based on the structural features of LTR-III, three putative binding sites could be proposed: (1) the loop duplex region (G5-C13); (2) the G-tetrad at the base of the G-quadruplex (G17-G21-G25-G28 flanked by the A22-C23-T24 lateral loop); and (3) the G-tetrad at the quadruplex–duplex junction (G2-G15-G19-G26), including the flanking junction bases (G3-A4-T14) within the cavity between the two distinct DNA sections (Figure 1a). We recognized that this specific G4/duplex junction offers a distinct ligand binding site (Figure 1b) compared to the isolated G-tetrad flanked by a classical lateral loop (Figure 1c).^35^

Herein, we report a proof-of-concept study, whereby we introduce a pyridinium-functionalized cyclic-bridged azobenzene ligand **1** (Figure 1d) that, in its *trans* elongated conformation, acts as an efficient and selective G4/duplex junction-targeting photoswitch. To the best of our knowledge, this study represents the first example of a photoresponsive ligand reversibly targeting this type of junction. A multidisciplinary approach that combines binding studies, NMR spectroscopy, and molecular modeling was used to identify the binding mode and strength of the interaction. In particular, we achieved the fully reversible switching of ligand binding affinity between the *cis* and *trans* forms in the presence of G4 DNA, mediated by low-intensity blue (405 nm) and green (520 nm) light under physiological conditions, with a hitherto unrealized ∼50-fold difference in binding affinity between the two photoisomers. To the best of our knowledge, this difference in G4 DNA binding activity between two photoisomers of the same compound is the highest reported so far. Computational and structural methods demonstrate that the elongated *trans* conformation of the ligand indeed targets the unique G4/duplex junction in the (3 + 1) hybrid LTR-III G4/duplex rather than the bare G-tetrad. Together, our results reveal that the structural switch of the ligand by an external stimulus, such as light, can be used to control binding affinity and mode of binding in a fully reversible manner with exquisite selectivity under conditions relevant to biological applications, without the need to preincorporate photoresponsive functionality in the biomolecule.

## 2 Results and Discussion

Ligand design of the target compound (**1**) was informed from our previous studies of *N*-methylated pyridinium compounds as photoresponsive G4 binding ligands,^25,41^ coupled with the many advantages of novel cyclic-bridged azobenzene scaffolds. In particular, cyclic-bridged azobenzene possesses excellent and well-characterized photophysical properties, demonstrating visible-light-controlled photoisomerizations in both directions over many cycles without photofatigue.^19,42–44^ Moreover, the rigid nature of the fused cyclic system confers an additional advantage compared to the more flexible parent azobenzene, as several studies demonstrate that increased ligand rigidity confers superior G4 binding properties.^45^ In addition, the very unusual twisted geometry of the *trans* form of the scaffold represents an exceptionally unique chemotype for DNA recognition, which has not yet been explored: of the hundreds of G4 ligands reported to date, to the best of our knowledge, there is no example of a G4 binding ligand that possesses this type of molecular geometry. Thus, we envisaged that the facile incorporation of positively charged pyridinium units would confer DNA binding properties to the parent scaffold and, in its “active” elongated state, which is closer to a planar conformation compared with the bent *cis* isomer, selectively target DNA G4 structures.

### 2.1 Synthesis

The synthetic route to the cyclic azobenzene ligand *cis*-**1** is depicted in Scheme 1. *Cis*-2,9-dibromo-11,12-dihydrodibenzo­[*c*,*g*]­[1,2]-diazocine **2** was prepared using an established procedure with minor modifications.^44^ Briefly, Suzuki coupling of 4-pyridinylboronic acid with the bisbrominated cyclic azobenzene **2** afforded compound **3** in 99% yield. Finally, alkylation with iodomethane provided the target compound *cis*-**1** in an excellent yield of 75%. All compounds were fully characterized with standard spectroscopic and analytical techniques (ESI).

**Scheme 1:**
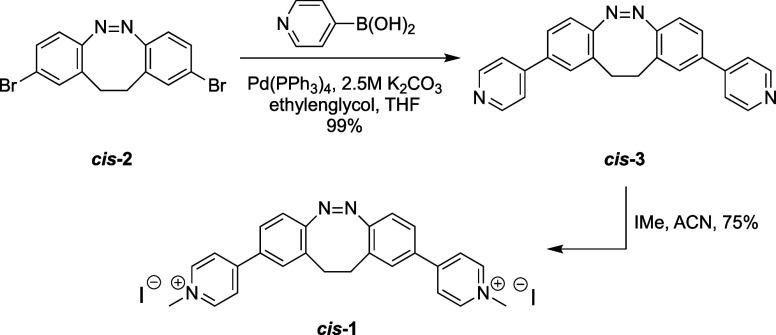
Synthesis of the Bridged Azobenzene *cis*-**1**

### 2.2 Ligands Cis-1/Trans-1 Are Reversely Photoswitchable under Physiological Conditions without Photofatigue

With ligand *cis*-**1** in hand, we initially focused our efforts on evaluating its photophysical properties using UV–Vis spectroscopy (Figures 2 and S1) since the nature of the cationic *N*-methylated pyridinium groups on the photochemistry of the cyclic azobenzene was unknown. In conditions relevant to G4 folding (namely, 20 mM potassium phosphate buffer (pH 7.0) containing 70 mM KCl), the thermodynamically stable *cis* isomer exhibits an absorption maximum at 394 nm corresponding to a π–π* transition (compared to 404 nm in the corresponding unsubstituted cyclic azobenzene).^44^ Irradiation close to this maximum (λ = 405 nm, low-intensity blue light) induced rapid spectral changes (reaching a photostationary state after approximately 4.5 min, *k*_rel_ = (15.37 ± 0.43) × 10^–3^ s^–1^), namely, a decrease in absorption at 400 nm and the emergence of a red-shifted maximum at 480 nm, corresponding to a n−π* transition of the elongated *trans* isomer (Figures 2a and S1).

**Figure 2:**
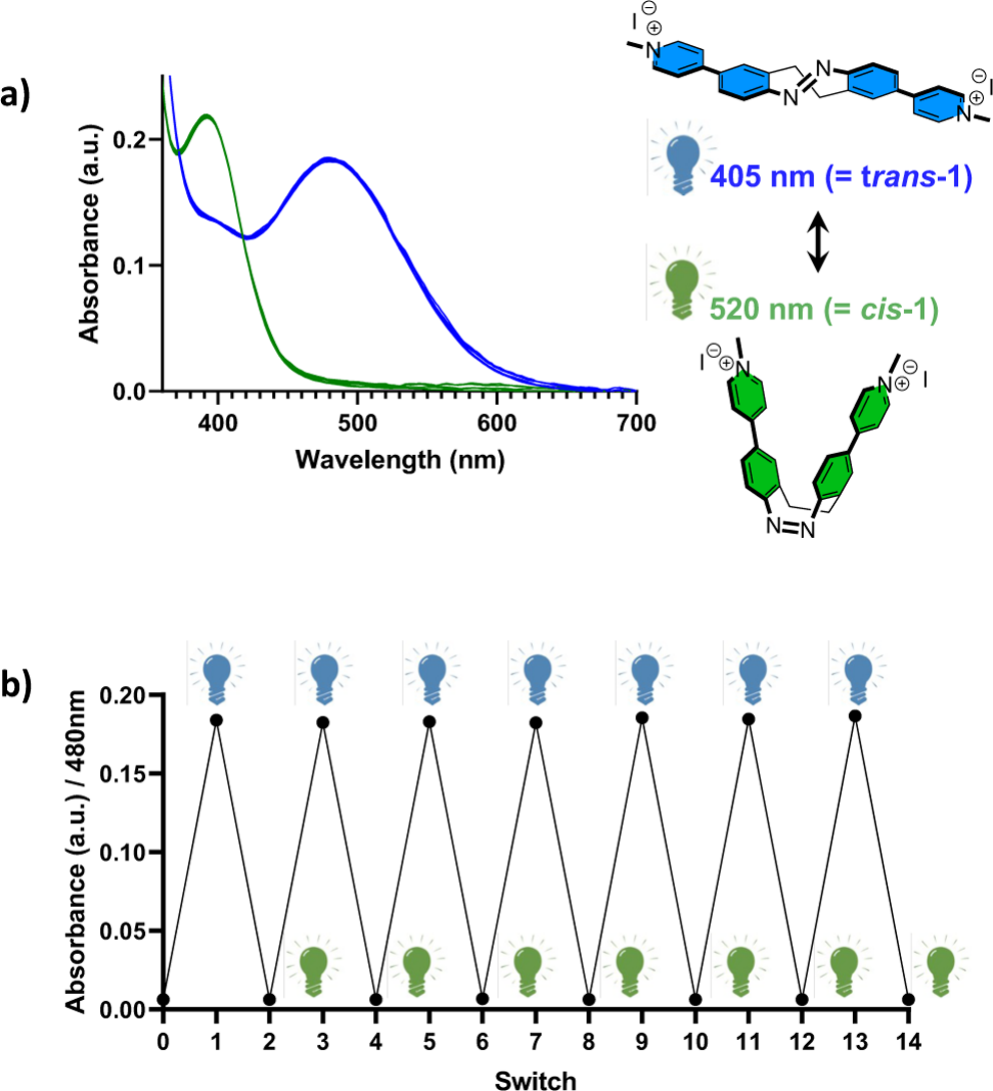
(a) Photoswitching between *cis*-**1**/*trans*-**1** states and (b) reversible switching over several cycles monitored by absorbance at 480 nm, monitored by UV/visible spectroscopy. [Ligand] = 100 μM, 20 mM KPhos buffer (pH 7.0) containing 70 mM KCl, room temperature (20 °C).

Next, we investigated the reversibility of the system both under photochemical conditions and at room temperature in the dark (thermal conditions). The relative rates of isomerization under otherwise comparable experimental conditions are summarized in Figure S1. The initial *cis* state of the molecule was recovered both under photochemical (λ_irr_ = 520 nm) and thermal conditions, although the back-isomerization was significantly slower under thermal conditions (first-order decay half-lives were 42 s and 5.4 min, respectively). Due to the rapid thermal relaxation of the “active” *trans*-**1** isomer, studying its binding by NMR at ambient temperature was not feasible, as it quickly reverted to the “less active” *cis*-**1** on the time scale of the experiment (*vide infra*). To differentiate the binding properties of the two isomers, we conducted the study of the reversibility of the system at a lower temperature (5 °C) in the dark (Figure S1d). Unlike at room temperature, the back-isomerization at 5 °C is significantly slower with a first-order decay half-life of 17.4 min, allowing the NMR experiment. However, UV–Vis and CD experiments involving the *trans* form were conducted at ambient temperature under continuous light irradiation, with the light source positioned 10 cm from the sample, to suppress thermal back-isomerization.

Finally, we examined the photostability of the system over 14 switches between *cis* and *trans* conformations (Figure 2b) by alternately irradiating the system with low-intensity blue (λ = 405 nm) and green light (λ = 520 nm). As shown in Figure 2, the isosbestic points are preserved throughout the experiment, and the respective UV/visible spectra are perfectly superimposable, indicating no photodecomposition under the reaction conditions.

### 2.3 *Trans*-1 Binds LTR-G4 with 50-Fold Greater Affinity Than the *Cis* Photoisomer and Allows Full Control of Binding Affinity *In Situ*

Having demonstrated the robust and reversible *cis–trans* photoisomerization of ligand **1** under relevant conditions to G4 DNA folding, we initially studied the binding to the TLR-III G4 sequence. UV/visible titration studies (Figure 3) revealed an association constant (*K*_a_) of 5.75 × 10^5^ M^–1^ for the *trans* isomer, while the affinity of the bent *cis* isomer for the same G4 was ∼50-fold lower, *K*_a_ = 0.11 × 10^5^ M^–1^. This difference in DNA binding activity between two photoisomers of the same compound is, to the best of our knowledge, significantly higher than those previously reported to date (∼10-fold between the 405 nm illuminated *trans* isomer and the nonilluminated *cis* isomer for the DNA G4MYC structure).^46^ Moreover, *trans*-**1** showed hypochromic and bathochromic shifts upon ligand binding, suggesting end-stacking ligand binding modes, where the excitation energy of the π–π* transition band is lowered by the interactions of the ligand chromophores with the G-tetrad.^47^ UV/visible titration experiments using a duplex DNA model (ds26) revealed comparable binding affinities for both *cis* (*K*_a_ = 2.53 × 10^3^ M^–1^) and *trans* (*K*_a_ = 2.44 × 10^3^ M^–1^) isomers (Figure S2a), which are 1 and 2 orders of magnitude lower, respectively, than those observed for the HIV LTR-III G4 structure. These results strongly suggest that the *trans* isomer selectively binds the G4 region of HIV LTR-III, rather than the duplex stem.

**Figure 3:**
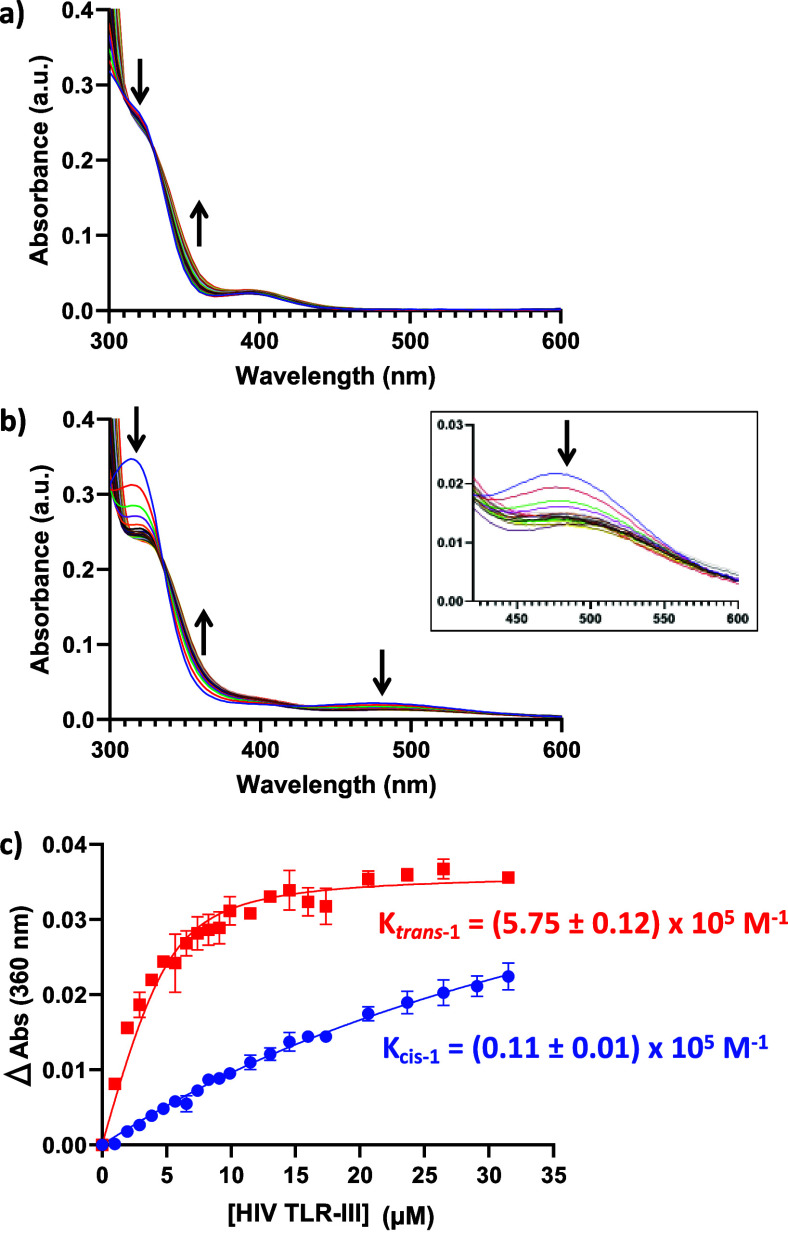
UV/visible titration studies of (a) *cis*-**1** and (b) *trans*-**1** with LTR-III (K^+^) and (c) binding isotherms in 20 mM KPhos buffer (pH 7.0) containing 70 mM KCl, room temperature (20 °C).

### 2.4 Fully Reversible Switching of *Cis*-1/*Trans*-1 Binding in the Presence of LTR-III G4 DNA

In order to demonstrate that the binding affinity of *cis*-**1**/*trans*-**1** could be regulated in situ in the presence of G4 DNA, we turned to circular dichroism (CD) spectroscopy. Under the experimental conditions reported by Phan and co-workers,^28^ the formation of the duplex/G4 hybrid is observed by two positive CD signals at 269 and 285 nm. Based on previous investigations,^48,49^ it is likely that the duplex stem contributes to the signal at ∼270 nm, while the G4 structure contributes to the signal at ∼290 nm. A strong negative band at 240 nm is also observed, another common spectral feature of G4 DNA secondary structures.^48,49^ Upon titrating the DNA structure with the “less active” bent *cis*-**1**, virtually, no changes in the CD signal are observed either in the DNA spectral region (λ < 320 nm) or in the ligand-only region (λ > 320 nm), where certain binding modes may be evidenced by the appearance of an induced CD in the achiral ligand upon binding to the chiral DNA structure (Figure 4a).^50^ In the case of the “active” elongated *trans*-**1**, however, a strikingly different behavior is observed (Figure 4b). As the ligand concentration is increased, a hyperchromic shift in the positive CD signal of the DNA structure is clearly observed, and a strong CD signal is induced into the ligand (specifically, a negative band at λ = 330 nm and a positive band at λ = 480 nm). This provides compelling evidence for the association of the ligand with the chiral DNA and, together, the CD results observed for the nonilluminated *cis* and the 405 nm illuminated *trans* isomers support the results of the UV/Vis titration studies that suggest two distinct binding modes and affinities of the two photoisomers for the DNA structure. Moreover, NMR experiments (vide infra) provided further evidence to support the ability of elongated *trans*-**1** to target the G4 region of the DNA structure.

**Figure 4:**
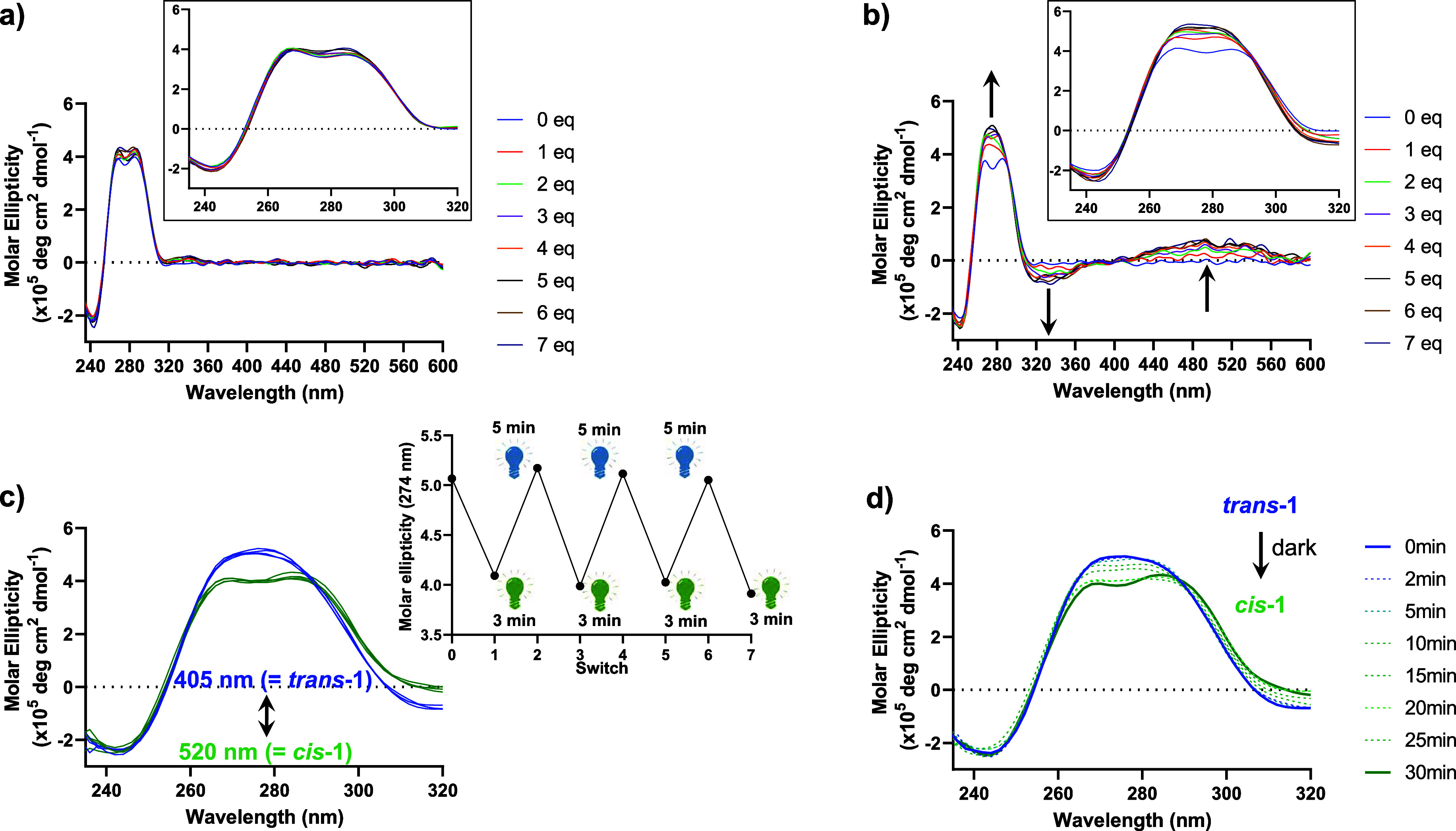
CD titrations of (a) *cis*-**1** and (b) *trans*-**1** to LTR-III (including induced CD); (c) reversible in situ regulation of ligand binding proved by CD spectroscopy (the inset shows the reversible switching over several cycles by monitoring at 274 nm); (d) thermal relaxation and unbinding of the ligand in the dark, in 20 mM KPhos buffer (pH 7.0) containing 70 mM KCl, 20 °C.

Importantly, we found that the ligand binding and dissociation from the LTR-III G4 could be controlled reversibly by alternate irradiation with low-intensity blue (λ = 405 nm) and green light (λ = 520 nm), with no evidence of photofatigue after seven switches, and no disruption of the overall DNA structure was observed (Figure 4c). Moreover, in agreement with the preliminary experiments that demonstrated the back-isomerization of *trans* to *cis* conformation, we observed the dissociation of the ligand from the LTR-III structure under dark conditions triggered by the reversion of the “active” *trans* isomer to the “less active” *cis* form (Figure 4d). These results are significant as they demonstrate that the presence of the DNA structure does not adversely affect the photochemical properties of the ligand and that its activity can be controlled in situ. Control experiments demonstrated that neither *trans*-**1** nor *cis*-**1** bound a double-stranded DNA model (ds26), suggesting that the ligand indeed targets the G4 region of the structure and not the duplex stem (Figure S2), as previously observed by UV/visible titration.

### 2.5 Ligand Trans-1 Specifically Targets the G4/Duplex Junction

Based on the encouraging results observed for the LTR-III G4 in the UV/visible titration and CD studies, we chose to investigate the binding of *cis*- and *trans*-**1** to this DNA sequence in greater detail using a combination of ^1^H NMR spectroscopy and enhanced molecular dynamics (MD) simulations. Previous work by our group has identified that this combination of theoretical and experimental approaches is well-suited to understanding the different binding modes of G4-DNA-targeting molecules.^25,27,41^ In particular, we were curious to confirm that the elongated *trans* conformation of the novel ligand indeed targets the G4/duplex junction in LTR-III and not the duplex stem or lower G-tetrad.

#### 2.5.1 ^1^H NMR Spectroscopy

To further probe the different binding properties of *cis*- and *trans*-isomers, we turned to ^1^H NMR spectroscopy to obtain more detailed structural information about the binding interactions. The aforementioned fast thermal relaxation of the “active” *trans*-**1** prevented us from studying the binding by NMR at ambient temperature because the ligand reverts to the “less active” *cis*-**1** too quickly on the time scale of the NMR experiment. Therefore, we studied the ligand binding for both isomers at a lower temperature (5 °C) in order to discriminate between their different binding properties. In each case, ligand aliquots were added, and the spectral changes of the G4 Hoogsteen and duplex Watson–Crick resonances were monitored (Figures 5b, S3 and S4).

**Figure 5:**
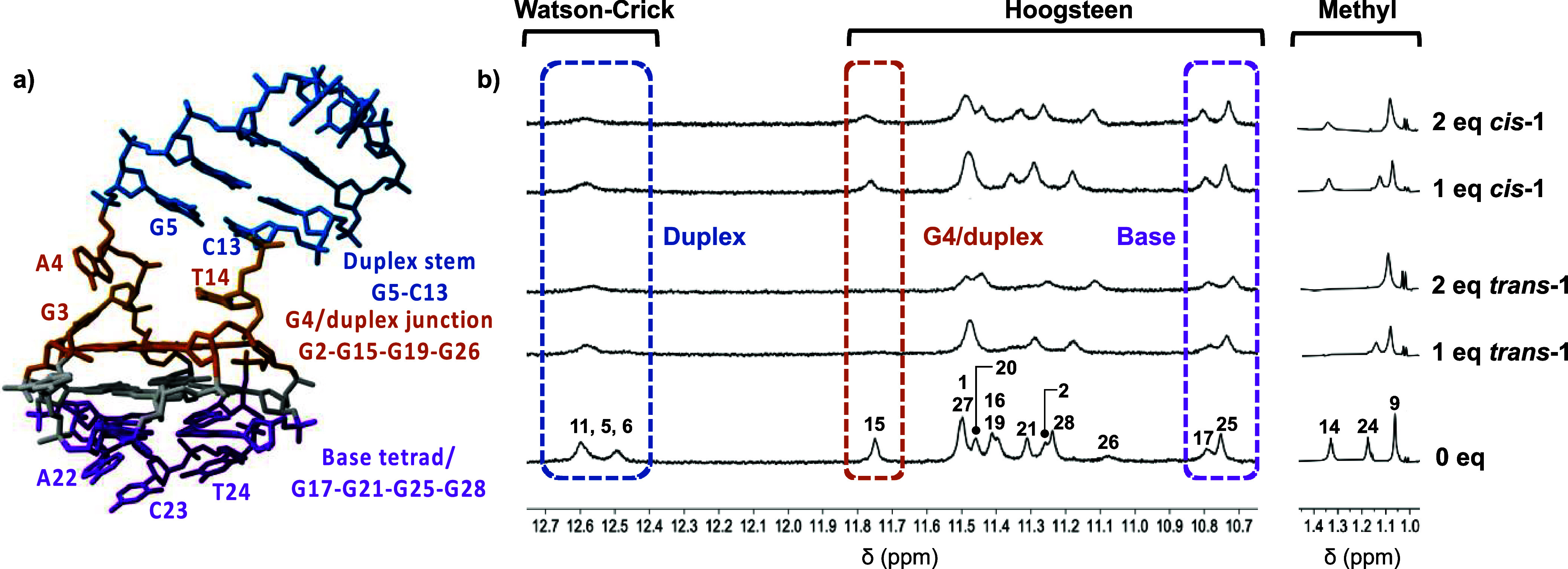
(a) Schematic structure of LTR-III showing potential ligand binding sites (base tetrad, purple; duplex stem, blue; G4/duplex junction, orange) and (b) stacked ^1^H NMR of LTR-III G4 imino and thymine methyl regions in NMR titration studies at 5 °C in a 20 mM KPhos buffer (pH 7) containing 70 mM KCl and 10% D_2_O with *cis*-**1** and *trans*-**1**.

In the case of the “active” elongated *trans*-**1**, strong spectral changes are observed in the G4 region of the DNA structure. Specifically, resonances corresponding to the top G-tetrad at the duplex/quadruplex junction (G15 and G19) broaden considerably and are undetectable following the addition of 1 equiv of the ligand. This behavior reflects slow-to-medium exchange on the NMR time scale, indicative of a strong binding event, where the dissociation rate of the complex is slow in comparison to the difference in frequencies between the free and bound resonances.^51,52^ Meanwhile, resonances corresponding to the lower G-tetrad are comparatively unperturbed (G17, G28, G25, and G21) and remain visible even following the addition of 2 equiv of the ligand. These results demonstrate that in the closer planar *trans* conformation, the ligand is able to efficiently stack with the top G-tetrad, between the G4/duplex junction, while it does not have strong affinity for the lower G-tetrad. Moreover, the T4 thymine methyl resonance (1.5 ppm), located at the quadruplex-duplex junction, quickly broadens out and disappears upon titrations with 405 nm illuminated *trans*-**1**. In comparison, the other thymine signals T9 and T24 (1–2 ppm), which are loop residues, remain sharp (Figures 5b and S5).

Turning attention to the bent *cis* isomer, it is clearly observed that the G4 signals corresponding to the G4/duplex junction, e.g., G15 (top), remain distinct during the titration, indicating comparatively lower affinity to this region of the structure in comparison to the *trans* conformation. Indeed, only minor line broadening is observed, along with some gradual chemical shift changes for certain resonances, most obviously, G16, G19, and G28. This phenomenon is indicative of fast exchange on the NMR time scale and reflective of weak binding, where the low affinity leads to fast dissociation of the bound species. While perturbations of the observable Watson–Crick resonances are observed for both *trans* and *cis* isomers, it should be noted that the concentrations required for NMR experiments (185 μM) are significantly above the *K*_d_ values (1.75 and 90.9 μM for *trans*-**1** and *cis*-**1**, respectively) observed in the UV/visible titration experiments and much higher than the concentrations required for CD studies. Therefore, much weaker nonspecific duplex binding events may be observed, though these are unlikely to be relevant under appropriate conditions for biological and nanotechnological applications, which normally employ micro- or submicromolar level concentrations of the DNA and ligands. Indeed, a control CD experiment showed no binding of either ligand to a duplex DNA model under physiologically relevant concentrations (Figure S2, ESI).

#### 2.5.2 Molecular Dynamics Simulations

Molecular docking calculations, followed by MD simulations, were performed to assess the stability of the binding of *trans*-**1** and *cis*-**1** to HIV-1 LTR-III (see ESI for full details). Initially, docking calculations^53^ (guided by the NMR chemical shifts observed experimentally) were used to identify potential binding modes of *trans*-**1** and *cis*-**1** when bound to HIV-1 LTR-III (Figure 6a–d). The binding site for the ligands is located around the G5, T14, and G15 region, as shown in the NMR chemical shifts. Ten simulations, each 200 ns, were performed for the DNA:*trans*-**1** and DNA:*cis*-**1** complexes (Figures S5 and S6). The time evolution of the root-mean-square deviations (RMSD) for the *trans*-**1** and *cis*-**1** complexes was used to evaluate the stability of DNA (Figures S7 and S8), and the lowest energy binding pose was identified from docking (Figures 6 and S9). In all models, *cis*-**1** and *trans*-**1** remained bound to the region located above the top G-quadruplex plane (formed by G2, G15, G19, and G26). While the bent *cis*-**1** was located nearer to the duplex region, the elongated *trans*-**1** adjusted its position to get closer to the duplex–quadruplex junction (even embedding itself into that junction in some of the simulations) (Figure S10 (models 1–4 and models 6–10) and S11 (models 1–3 and models 5–10)). This observation suggests that the *trans* isomer binds preferentially at the junction, whereas the *cis* conformation favors the duplex part, which is in accordance with the NMR observations.

**Figure 6:**
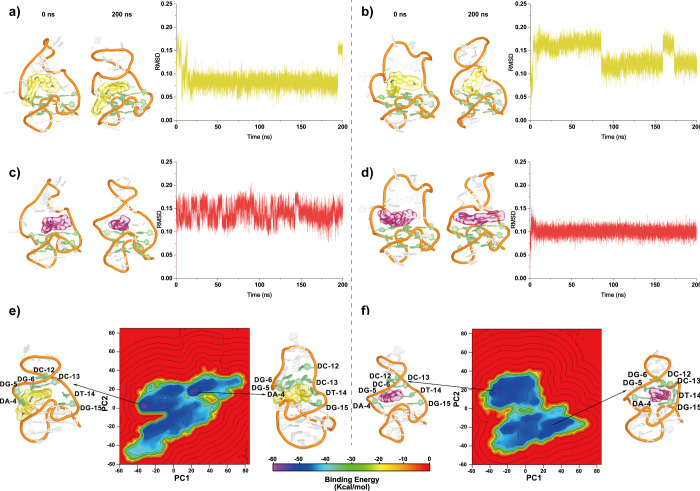
Examples of the behavior of the DNA–ligand complexes during the MD simulations and PCA-MMPBSA landscape. The behavior for models 3 and 10 is shown. The structures represent the starting and final conformations after 200 ns for each *cis*-**1** (yellow sticks) and *trans*-**1** (red sticks) complexes, whereas the plots show the RMSD of the ligand relative to its starting conformation for (a) the complex between model 3 and *cis*-**1**; (b) the complex between model 10 and *cis*-**1**; (c) the complex between model 3 and *trans*-**1**; and (d) the complex between model 10 and *trans*-**1**. (e) Energy landscape determined using the MMPBSA energies calculated for the *cis*-**1** complex. (f) Combined binding energy landscape determined using the MMPBSA energies calculated for the *trans*-**1** complexes. In panels (e,f), the green sticks represent the binding pocket bases, whereas the yellow and red spheres show the *cis*-**1** and *trans*-**1** ligands, respectively. Note: For the *trans*-**1** systems (panel f), 1401 conformations out of 100,000 have binding energies < -55 kcal/mol, whereas for the *cis*-**1** complexes (panel e), only 1165 frames out of 100,000 show such strong binding. The conformations shown in panels (e,f) represent the centroid frames for the two lowest energy minima for each of *trans*-**1** and *cis*-**1**. These conformations were extracted to identify the ligand binding modes and the structural features of the binding pockets in each case.

To obtain further structural details on the binding of *trans*-**1** and *cis*-**1**, principal component analysis (PCA) of the binding pockets was performed using the C1’atom (the carbon atom linking the deoxyribose and base in the DNA) and the heavy atoms of the ligands (Figure S12a–e). This analysis showed a noticeable difference in the distribution of the pocket between *trans*-**1** and *cis*-**1**, with the two ligands exploring different regions of the conformational space. Nonetheless, there is some overlap between the distributions for the two complexes, implying that the binding modes of the ligands share some common structural features (Figure S12).

Clustering using Dbscan^54^ was performed to characterize the conformational landscape of the binding pockets in the presence of the *trans*-**1** and *cis*-**1** conformations. Four and seven clusters were found for the *trans*-**1** and *cis*-**1** complexes, respectively (these are represented by their centroid structure in Figure S12d–e). Notably, all centroid frames for the *trans*-**1** clusters show that the ligand is located at the junction region close to T14 and in contact with G15 (in line with the NMR data above). In contrast, the behavior of *cis*-**1** is more diverse, with four of the clusters (cluster (0–3)), showing one of the pyridine rings of the ligand partly inserted in the junction and the other around G5 and G6. In the remaining three clusters (4–6), the ligands are located away from the pocket in the junction region. This diversity of binding modes suggests less stable binding of *cis*-**1** to HIV-1 LTR-III compared to *trans*-**1**, which correlates well with the NMR shifts and UV/visible binding affinity data (Figures 3 and 5).

The MMPBSA (molecular mechanics energies combined with the Poisson–Boltzmann and surface area continuum solvation) approach was used to estimate the binding energy for all ten models for *trans*-1 and *cis*-1 complexes.^55^ As shown in Table S2, the average binding energies, calculated by aggregating data from all ten individual models for each system, indicate a mild preference for the *trans-* **1** isomer over *cis-* **1**, with a difference of 0.8 kcal/mol. Although small, this difference is statistically significant (see Table S3 for more details) and aligns with the experimental findings, indicating that *trans-* **1** preferentially binds to the LTR-III G4 structure.

Despite some exceptions, most individual models show more negative binding energies for *trans-* **1** compared to *cis-* **1**, reinforcing that the former generally exhibits higher binding affinity to HIV-1 LTR-III. However, when comparing only the lowest binding energies, for *cis-* **1** in model 2 (−48.49 kcal/mol) and *trans-* **1** in model 9 (−46.63 kcal/mol), the trend appears reversed, with *cis-* **1** showing stronger binding to the DNA. This discrepancy highlights the limitations of relying on a single model, as it may overlook structural variability and the broader conformational landscape. In this particular case, using only models 2 and 9 for *cis-* **1** and *trans-* **1**, respectively, represents just 10% of all available conformational data. Our results here underscore the importance of considering system dynamics and the impact of sampling on binding energy estimates. Unlike comparisons based solely on the most favorable binding models, analyzing average binding energies offers a more comprehensive view of the systems’ behavior, which in this case is consistent with experimental findings.

An energy landscape was constructed using PCA and the calculated MMPBSA energies to identify the structural features of the preferred binding modes for each ligand when bound to DNA (Figure 6e,f). Figure 6e,f shows that the binding of *trans*-**1** to the DNA is stronger. In the centroid frames of the *trans*-**1**:HIV-1 LTR-III complex, the ligand sits at the junction between the duplex and quadruplex regions of DNA, forming stable hydrogen bonds with T14 and G15. We observe that the ligand adopts different binding modes in the two centroid frames of the *cis*-**1** complex: in one frame, one of the pyridine rings is inserted directly into the junction; in the other, the azobenzene moiety is close to the junction. Despite these differences, in both frames, *cis*-**1** is always surrounded by G5 and G6 from the duplex region and is located far away from G15.

Our simulations show different binding mechanisms for *trans*-**1** and *cis*-**1**: the flat *trans*-**1** ligand presents a more rigid complex in the junction part, whereas the bent *cis* isomer shows an unstable complex in the duplex part involving the pyridine ring. These results demonstrate the potential application of the azobenzene ligand *trans*-**1** as a highly selective binding fragment in the junction part of mixed quadruplex and duplex sequences.

## 3 Conclusions

We introduce, to the best of our knowledge, the first class of photoswitchable G4 DNA ligands based on a cyclic azobenzene core with distinct binding modes and affinities to a G4/duplex junction in their two conformational states. We show that the elongated *trans* isomer of the *N*-methylated pyridinium diazocine ligand selectively targets the G4/duplex junction of the HIV-1 LTR-III sequence over its duplex DNA stem. We demonstrated the robust and fully reversible photoisomerization of the diazocine ligand at physiological pH and ionic strength and achieved a > 50-fold affinity difference between the *trans* and *cis* isomers as determined by UV/visible and CD titration studies. To date, this affinity difference is the highest reported for G4 DNA binding activity between two photoisomers of the same compound . CD experiments further demonstrated that the ligand binding and dissociation from LTR-III G4 can be controlled reversibly by alternate irradiation with low-intensity blue (λ = 405 nm) and green light (λ = 520 nm). Further evidence of the differential behavior of the *trans* and *cis* isomers was obtained by ^1^H NMR studies in combination with MD simulations. These experiments support the >50-fold preferential binding of the elongated *trans* conformation at the G4/duplex junction of the LTR-III sequence, whereas the bent *cis* isomer favors the duplex region. Our results demonstrate that the conformational switch of the ligand by an external stimulus, such as light, can be used to control binding affinity and mode of binding in a fully reversible manner without the need to preincorporate photoresponsive functionality in the biomolecule, thus mitigating virtually all limitations of previously reported systems. These results pave the way for the development of new photoswitchable G4 ligands that selectively target quadruplex–duplex junctions, a DNA motif which is gaining increasing attention as drug targets due to their frequent occurrence in genomic sequences, to probe further the role of transient G4 formation in cellular functioning, or even to control G4 supramolecular complexes for nanotechnological applications.

## 4 Materials and Methods

### 4.1 General

Reagents and solvents were purchased as reagent grade and used without further purification. Cis-2,9-dibromo-11,12-dihydrodibenzo­[c,g]­[1,2]­diazocine (cis-2)^44^ was prepared according to previously reported procedures. For column chromatography, silica gel 60 (230–400 mesh, 0.040–0.063 mm) was purchased from E. Merck. Thin-layer chromatography (TLC) was performed on aluminum sheets coated with silica gel 60 F254, purchased from E. Merck, and visualized by UV light. NMR spectra were recorded on a Bruker AC 400 with solvent peaks as a reference. ^1^H and ^13^C NMR spectra were obtained for solutions in CDCl_3_ and DMSO-d_6_. All the assignments were confirmed by one- and two-dimensional NMR experiments (DEPT, COSY, HSQC, and HMBC). Mass spectra were obtained by the University of Bristol mass spectrometry service by electrospray ionization (ESI).

### 4.2 Chemical Synthesis

#### 4.2.1 *Cis*-2,9-Di­(pyridin-4-yl)-11,12-dihydrodibenzo­[*c*,*g*]­[1,2]­diazocine (*cis*-3)

A suspension of *cis*-2,9-dibromo-11,12-dihydrodibenzo­[*c*,*g*]­[1,2]­diazocine (*cis*-2) (400 mg, 1.10 mmol), Pd­(PPh_3_)_4_ (128 mg, 0.11 mmol), ethylene glycol (1 drop), and 4-pyridinylboronic acid (450 mg, 3.30 in a mixture of THF (15 mL) and aq. 2.5 M K_2_CO_3_ (4 mL)) was bubbled with N_2_ for 10 min. The resulting solution was heated to 70 °C overnight. After the mixture was cooled to room temperature, water was added. The aqueous layer was extracted with DCM (×2), and the combined organic extractions were dried over MgSO_4_, filtered, and concentrated in vacuo. The residue was purified by flash silica chromatography (DCM/MeOH, 40:1), affording compound *cis*-**3** (395 mg, 99%) as a yellow amorphous solid. ^1^H NMR (400 MHz, CDCl_3_) δ 8.60 (d, *J* = 6.2 Hz, 4H, H-12), 7.45 (dd, *J* = 8.1, 1.9 Hz, 2H, H-8), 7.40 (d, *J* = 6.2 Hz, 4H, H-11), 7.29 (d, *J* = 1.9 Hz, 2H, H-6), 7.01 (d, *J* = 8.1 Hz, 2H, H-9), 3.11 (m, 2H, H-5), 2.92 (m, 2H, H-5); ^13^C NMR (101 MHz, CDCl_3_) δ 156.0 (C-3), 150.4 (C-12), 147.1 (C-10), 137.1 (C-7), 128.9 (C-4), 128.5 (C-6), 125.7 (C-8), 121.4 (C-11), 120.0 (C-9), 31.9 (C-5); ESI-HRMS for C_24_H_19_N_2_ [M + H]^+^ calcd: 363.1604, found: 363.1609.

#### 4.2.2 *Cis*-4,4′-(11,12-Dihydrodibenzo­[*c*,*g*]­[1,2]­diazocine-2,9-diyl)­bis­(1-methylpyridin-1-ium) iodide (*cis*-1)

To a solution of compound *cis*-**3** (50 mg, 0.14 mmol) in acetonitrile (4 mL) was added methyl iodide (35 μL, 0.55 mmol). The solution was stirred in a sealed vessel at 50 °C overnight. The generated solid was filtered and washed with ether. Following filtration, compound *cis*-**1** (67 mg, 75%) was obtained as a yellow powder. ^1^H NMR (400 MHz, DMSO-*d*_6_) δ 8.97 (d, *J* = 6.9 Hz, 4H, H-12), 8.43 (d, *J* = 7.1 Hz, 4H, H-11), 7.98–7.92 (m, 4H, H-6, H-8), 7.24 (d, *J* = 8.7 Hz, 2H, H-9), 4.29 (s, 6H, H-13), 3.14–2.99 (m, 4H, H-5); ^13^C NMR (101 MHz, DMSO-*d*_6_) δ 157.6 (C-3), 152.9 (C-10), 145.5 (C-12), 132.3 (C-7), 130.0 (C-6), 129.2 (C-4), 126.9 (C-8), 123.9 (C-11), 119.9 (C-9), 47.1 (C-13), 30.7 (C-5); ESI-HRMS for C_26_H_24_IN_4_^+^ [M]^+^ calcd: 519.1040, found: 519.1052.

### 4.3 UV–Visible Spectroscopy

UV spectra were recorded on a Thermo Scientific BIOMATE 3S UV–Vis spectrophotometer at ambient temperature. Measurements were taken in a 3 mL quartz cuvette with a path length of 10 mm. The UV–visible spectra were recorded between 700 and 300 nm and baseline-corrected for the buffer used.

### 4.4 Photoirradiation Experiments

Samples of *cis*-1 and *trans*-1 (100 μM in 20 mM potassium phosphate (KPhos) buffer (pH 7.0) and 70 mM KCl) were irradiated with monochromatic light at 405 and 520 nm, respectively. The irradiation was performed in a 3 mL quartz cuvette containing a magnetic stirrer and a total volume of 2 mL solution. The irradiation sources were collimated laser diode modules (ThorLabs, CPS405 and CPS520, 4.5 mW, elliptical beam). The photoisomerization was followed by recording the UV–visible spectra at appropriate time points.

For the kinetic experiments, samples of 1 (10 μM) and the appropriate DNA sequence (100 μM by strand) were exposed to ambient room light under otherwise identical conditions. The irradiation was performed in a 3 mL quartz cuvette containing a total volume of 1.5 mL of solution. The buffer used was 20 mM potassium phosphate (pH 7.0) and 70 mM KCl.

UV–Vis and CD experiments involving the *trans* form were conducted at ambient temperature under continuous light irradiation, with the light source positioned 10 cm from the sample to suppress thermal back-isomerization.

For photoirradiation experiments conducted in the presence of oligonucleotides and followed by NMR spectroscopy to ensure even irradiation of the sample, the solution (600 μL) was transferred to a 1 mL quartz cuvette containing a magnetic stirrer, irradiated at the appropriate wavelength for the time specified, and then transferred back to the NMR tube for analysis.

Apparent association (*K*_a_) or dissociation (*K*_d_) constants for *cis*-1 and *trans*-1 were determined through UV–Visible spectroscopy titration experiments. The raw spectra were recorded as described in Section 3. The concentration of the ligand was fixed at 10 μM in a constant volume of 1.5 mL of buffer. The oligonucleotide sequences used were HIV LTR-III (5′-GGGAGGCGTGGCCTGGGCGGGACTGGGG-3′) and ds26 (5′-CAATCGGATCGAATTCGATCCGATTG-3′). The oligonucleotide was purchased from Eurogentec (Belgium), purified by HPLC, and delivered dry. Oligonucleotide concentration was determined by UV absorbance using a NanoDrop 2000 Spectrophotometer from Thermo Scientific. The buffer used was 20 mM potassium phosphate (pH 7.0) and 70 mM KCl or 100 mM pH 7.4 KPhos (ds26). During the titration, aliquots of the sample were removed and replaced with aliquots of oligonucleotide to give the required titration points (from a 100 μM stock solution in appropriate buffer containing a 10 μM ligand to maintain a constant ligand concentration). NB: The oligonucleotide solution was annealed by heating to 90 °C for 2 min and then cooling on ice prior to the addition of the ligand (to avoid annealing in the presence of the ligand). Following the addition, the solution was mixed thoroughly, and the UV–Visible spectrum was acquired immediately. Data were fitted to an independent-and-equivalent-site binding model (eq 1) using Prism 7 software, a full derivation of which is provided by (among others) Thordarson,^56^ adapted to an independent and equivalent site model by (among others) Buurma and co-workers.^57^ The stoichiometry of the complex (*N*) was chosen as the lowest integer value that provided a satisfactory fit, *R* ^2^ > 0.97 (*N* = 2, in all cases). The data presented in Figure 3 show the average values obtained from two independent experiments.

### 4.5 Circular Dichroism Titrations

Circular dichroism (CD) titrations were recorded by using a Jasco J-815 Spectrometer fitted with a Peltier temperature controller. Measurements were taken in a quartz cuvette with a path length of 5 mm at 20 °C, using a scanning speed of 1000 nm/min at 1 nm intervals with a bandwidth of 1 nm. The CD spectra were recorded between 600 and 220 nm and baseline-corrected for the buffer used. The oligonucleotide sequences used were HIV LTR-III (5′-GGGAGGCGTGGCCTGGGCGGGACTGGGG-3′) and ds26 (5′-CAATCGGATCGAATTCGATCCGATTG-3′). The oligonucleotides were purchased from Eurogentec (Belgium), purified by HPLC, and delivered dry. Oligonucleotide concentrations were determined by UV absorbance using a NanoDrop 2000 Spectrophotometer from Thermo Scientific. The oligonucleotide was annealed before use by heating for 2 min at 90 °C and then placed immediately into ice. The oligonucleotide was at a concentration of 5 μM, which gave an OD of 1, and the buffer used was 20 mM potassium phosphate, pH 7.0, and 70 mM KCl (HIV TLR-III) or 100 mM pH 7.4 KPhos (ds26). The ligand was added by an aliquot from a 1 mM stock solution in the appropriate buffer (containing 10% DMSO to ensure solubility). The reported spectrum for each sample represents the average of 3 scans. Data processing was carried out using Prism 7 with an 8-point second-order smoothing polynomial applied to all spectra. Observed ellipticities were converted to mean residue ellipticity (θ) = deg cm^2^ dmol^–1^ (molar ellipticity).

### 4.6 NMR Spectroscopy Titrations

^1^H NMR spectra of G-quadruplex sequences were recorded at 278 K^58^ using a 600 MHz Varian VNMRS spectrometer equipped with a triple resonance cryogenically cooled probe head. The oligonucleotide sequence used was HIV LTR-III (5′-GGGAGGCGTGGCCTGGGCGGGACTGGGG-3′). Samples of oligonucleotide were dissolved in 90% H_2_O/10% D_2_O containing 20 mM potassium phosphate, pH 7.0, and 70 mM KCl. All experiments employed sculpted excitation with water suppression. The final NMR samples contained 600 μL of 185 μM oligonucleotide. Samples were annealed before use by heating for 2 min at 90 °C and then placed immediately into ice. Aliquots of ligands (10 mM in DMSO-*d*_6_) were added to the appropriate yield titration points, the sample was mixed thoroughly, and NMR spectra were recorded immediately after the addition of the ligand. Data were processed using MestReNova software (version 11.0.2). Resonances were assigned from data provided in the literature by Richter and co-workers.^28^ Photoirradiation of NMR samples was conducted using the protocol described in Section 4.4.

### 4.7 Molecular Dynamics Simulations

#### 4.7.1 Models for the Complexes between the G-Quadruplex Form of HIV-1 LTR and the Ligands

Molecular docking was used to build the complexes between the G-quadruplex form of HIV-1 LTR and the *cis*-**1** and *trans*-**1** ligands. For this, the HIV-1 G-quadruplex NMR structures (all ten models in the structure with the PDB code 6H1K)^28^ were used. The structures of *cis*-**1** and *trans*-**1** were optimized using the Gaussian16W^59^ program at the B3LYP-D3­(BJ)/def2-TZVP level of theory.^60,61^ AutoDockTools^53^ was used to prepare the protein and ligands and convert their PDB files into PDBQT format. All docking calculations were performed using AutoDock Vina (Version 1.2.0)^62^ (see the SI for more details). The DNA–ligand complexes were visualized using PyMOL (Version 2.5),^63^ with the ten lowest binding energy complexes (one per NMR model) being used as starting points for the MD simulations (see the SI for full details).

#### 4.7.2 Molecular Dynamics (MD) Simulations

Three systems were prepared: the DNA-*cis*-**1** and DNA-*trans*-**1** complexes, 10 different bound models were simulated for each ligand complex, along with a control system containing DNA without any bound ligands (see the SI for details). ACPYPE^64^ was used to obtain the parameters for the *cis*-**1** and *trans*-**1** ligands using the GAFF force field^65^ and AM1-BCC charge.^66^ The AMBER14sb_parmbsc1 ^67,68^ force field and TIP3P water model^69^ were used to describe the nucleic acids and water, respectively. All systems were solvated using a cubic box with a 1 nm solvent layer between the box edges and the solute surface. Two K^+^ ions were placed in the center of the three G-quadruplex planes, as described by Castelli et al.^70^ The systems were neutralized, and 100 mM concentration of K^+^ and Cl^–^ ions. The LINCS^71^ algorithm was used to constrain all bonds involving hydrogen atoms within the DNA and ligands, whereas the SHAKE algorithm^72^ was used for the water molecules. The integration step was 1.0 fs. Long-range electrostatic interactions beyond 1.2 nm were calculated using the smooth particle mesh Ewald (SPME) method.^73^ All systems were energy-minimized and simulated using the procedures and settings outlined in the SI. Full details of the subsequent analyses, including MMPBSA calculations and PCA, are provided therein.

## Supplementary Material



## Notes

Synthetic protocols and characterization data for all compounds, all biophysical characterizations using UV/visible, CD, and NMR, and computational studies (PDF)

## §

Instituto de Investigaciones Químicas (IIQ), CSIC – Universidad de Sevilla, Av. Américo Vespucio 49, Seville 41092, Spain
