# Supplementary material for: Bridged Azobenzene Exhibits Fully Reversible Photocontrolled Binding to a G‑Quadruplex DNA/Duplex Junction
Source: JACS Au. 2025 Aug 7;5(8):3846–57. doi: 10.1021/jacsau.5c00532 (PMC12381718; doi:10.1021/jacsau.5c00532)
Supplement: Supplementary file 1 [file au5c00532_si_001.pdf]

SUPPORTING INFORMATION

A bridged azobenzene exhibits fully-reversible photocontrolled binding to a G-quadruplex DNA/duplex junction

Javier Ramos-Soriano,*[a]† Y. Jennifer Jiang,[a] Bowen Deng,[a,b] Michael P. O´Hagan,[a] Aditya G. Rao,[b] Doudou Lu,[a] Susanta Haldar,[b] A. Sofia F. Oliveira,[b] Adrian J. Mulholland,*[b] and M. Carmen Galan*[a]

[a]School of Chemistry, University of Bristol, Cantock's Close, Bristol BS8 1TS, United Kingdom; [b]Centre for Computational Chemistry, University of Bristol, Cantock's Close, Bristol BS8 1TS, United Kingdom.

*E-mail: fj.ramos@iiq.csic.es, Adrian.Mulholland@bristol.ac.uk, m.c.galan@bristol.ac.uk

KEYWORDS: DNA nanodevices • photoswitch • azobenzene • G4/duplex DNA junction • supramolecular DNA interactions

1.	General…………………………………………………………………………...	S2
2.	Synthesis and characterization…………………………………………………	S2
3.	UV-visible spectroscopy……………………………………………………….	S6
4.	Photoirradiation experiments…………………………………………………..	S6
5.	Determination of apparent association or dissociation constants..…………………	S6
6.	Circular dichroism titrations…..………………………………………………..	S7
7.	NMR spectroscopy titrations………..………………………….……………...	S8
8.	Molecular dynamics simulations……………………………………………...	S14
9.	References………………………………………………………………………..	S26

1. General

Reagents and solvents were purchased as reagent grade and used without further purification. *cis*-2,9-dibromo-11,12-dihydrodibenzo[*c*,*g*][1,2]diazocine (***cis*-2**)[1] was prepared according to previously reported procedures. For column chromatography, silica gel 60 (230-400 mesh, 0.040-0.063 mm) was purchased from E. Merck. Thin Layer Chromatography (TLC) was performed on aluminium sheets coated with silica gel 60 F254 purchased from E. Merck, visualization by UV light. NMR spectra were recorded on a Bruker AC 400 with solvent peaks as reference. ^{1}H and ^{13}C NMR spectra were obtained for solutions in $CDCl_3$ and DMSO-d_6. All the assignments were confirmed by one- and two-dimensional NMR experiments (DEPT, COSY, HSQC and HMBC). Mass spectra were obtained by the University of Bristol mass spectrometry service by electrospray ionisation (ESI).

2. Synthesis and Characterization.

Synthesis of compound cis-1.

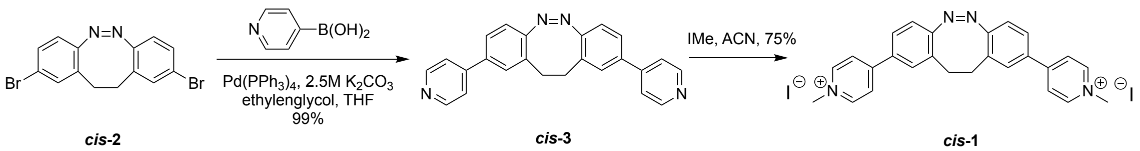

***cis*-2,9-di(pyridin-4-yl)-11,12-dihydrodibenzo[*c*,*g*][1,2]diazocine (*cis*-3)**

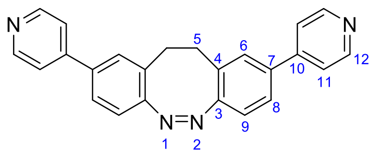

A suspension of *cis*-2,9-dibromo-11,12-dihydrodibenzo[*c*,*g*][1,2]diazocine (***cis*-2**) (400 mg, 1.10 mmol), Pd(PPh_3)$_4$ (128 mg, 0.11 mmol), ethyleneglycol (1 drop) and 4-pyridinylboronic acid (450 mg, 3.30 in a mixture of THF (15 mL) and aq. 2.5 M K_2CO_3 (4 mL) was bubbled with N_2 for 10 min. The resulting solution was heated at 70 °C overnight. After cooling to room temperature, water was added. The aqueous layer was extracted with DCM (x2) and the combined organic extractions dried over $MgSO_4$, filtered and concentrated *in vacuo*. The residue was purified by flash silica chromatography (DCM/MeOH, 40:1), afforded compound ***cis*-3** (395 mg, 99%) as a yellow amorphous solid. ^{1}H NMR (400 MHz, $CDCl_3$) δ 8.60 (d, J = 6.2 Hz, 4H, H-12), 7.45 (dd, J = 8.1, 1.9 Hz, 2H, H-8), 7.40 (d, J = 6.2 Hz, 4H, H-11), 7.29 (d, J = 1.9 Hz, 2H, H-6), 7.01 (d,

J = 8.1 Hz, 2H, H-9), 3.11 (m, 2H, H-5), 2.92 (m, 2H, H-5); ^{13}C NMR (101 MHz, $CDCl_3$) δ 156.0 (C-3), 150.4 (C-12), 147.1 (C-10), 137.1 (C-7), 128.9 (C-4), 128.5 (C-6), 125.7 (C-8), 121.4 (C-11), 120.0 (C-9), 31.9 (C-5); ESI-HRMS for $C_{24}H_{19}N_2$ $[M+H]^+$ calcd: 363.1604, found: 363.1609.

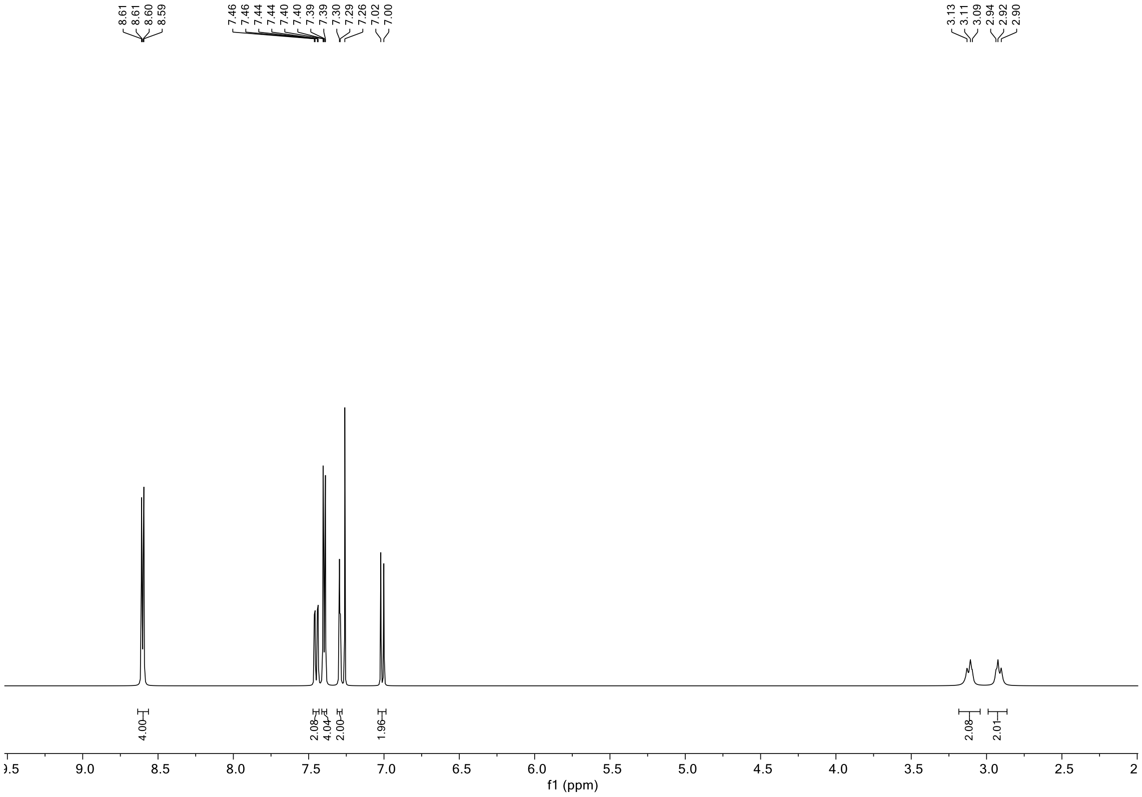

^{1}H NMR spectrum of compound ***cis*-3** ($CDCl_3$, 400 MHz).

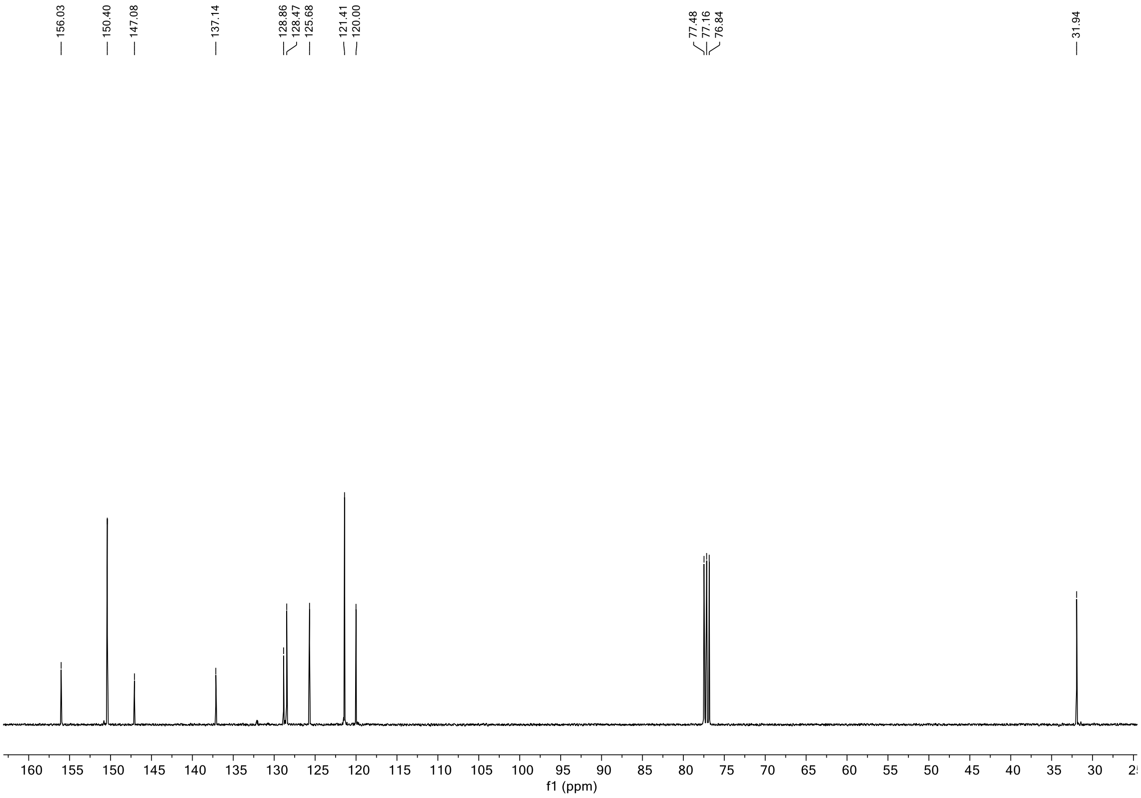

^{13}C NMR spectrum of compound ***cis*-3** ($CDCl_3$, 100 MHz).

***cis*-4,4'-(11,12-dihydrodibenzo[*c*,*g*][1,2]diazocine-2,9-diyl)bis(1-methylpyridin-1-ium) iodide (*cis*-1)**

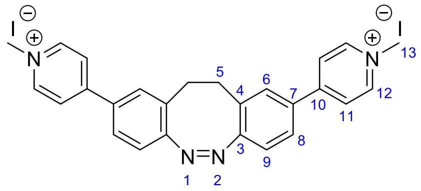

To a solution of compound ***cis*-3** (50 mg, 0.14 mmol) in acetonitrile (4 mL), methyl iodide (35 µL, 0.55 mmol) was added. The solution was stirred in a sealed vessel at 50 °C overnight. The generated solid was filtered and washed with ether. Following filtration, compound ***cis*-1** (67 mg, 75%) was obtained as a yellow powder. ^{1}H NMR (400 MHz, DMSO-d_6) δ 8.97 (d, J = 6.9 Hz, 4H, H-12), 8.43 (d, J = 7.1 Hz, 4H, H-11), 7.98 – 7.92 (m, 4H, H-6, H-8), 7.24 (d, J = 8.7 Hz, 2H, H-9), 4.29 (s, 6H, H-13), 3.14 – 2.99 (m, 4H, H-5); ^{13}C NMR (101 MHz, DMSO-d_6) δ 157.6 (C-3), 152.9 (C-10), 145.5 (C-12), 132.3 (C-7), 130.0 (C-6), 129.2 (C-4), 126.9 (C-8), 123.9 (C-11), 119.9 (C-9), 47.1 (C-13), 30.7 (C-5); ESI-HRMS for $C_{26}H_{24}IN_4^+$ $[M]^+$ calcd: 519.1040, found: 519.1052.

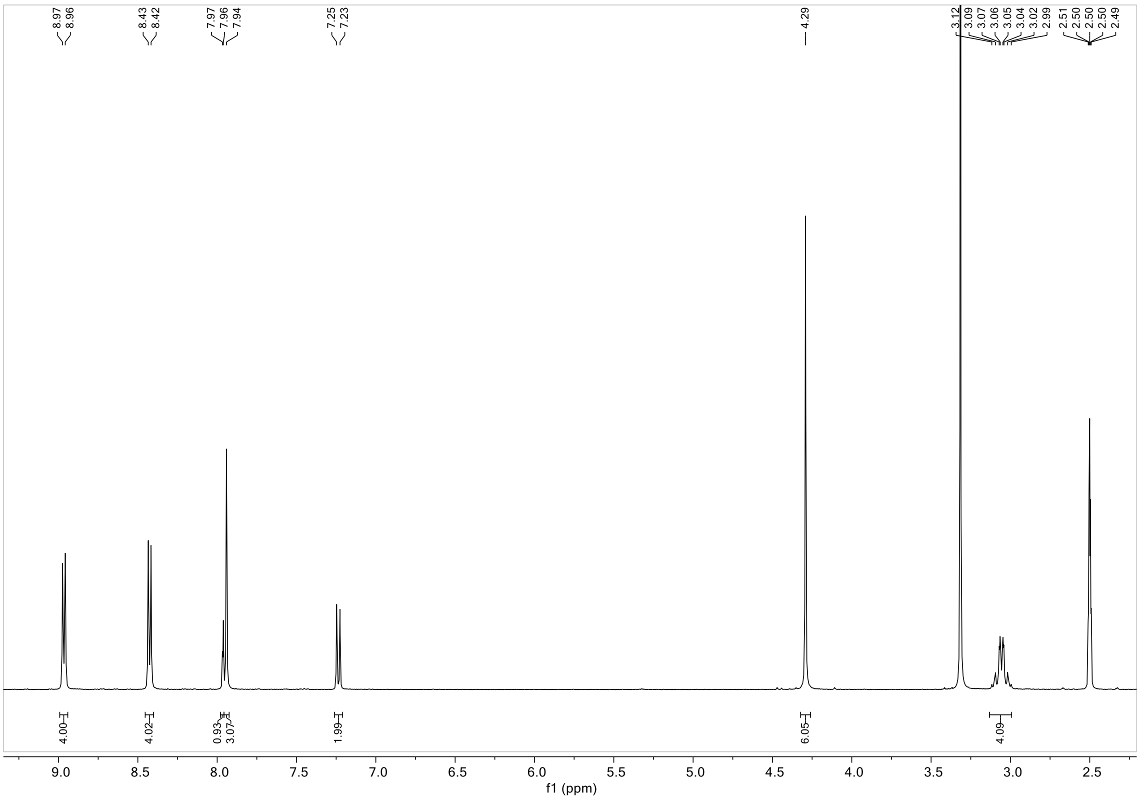

^1H NMR spectrum of compound ***cis*-1** (DMSO-d_6, 400 MHz).

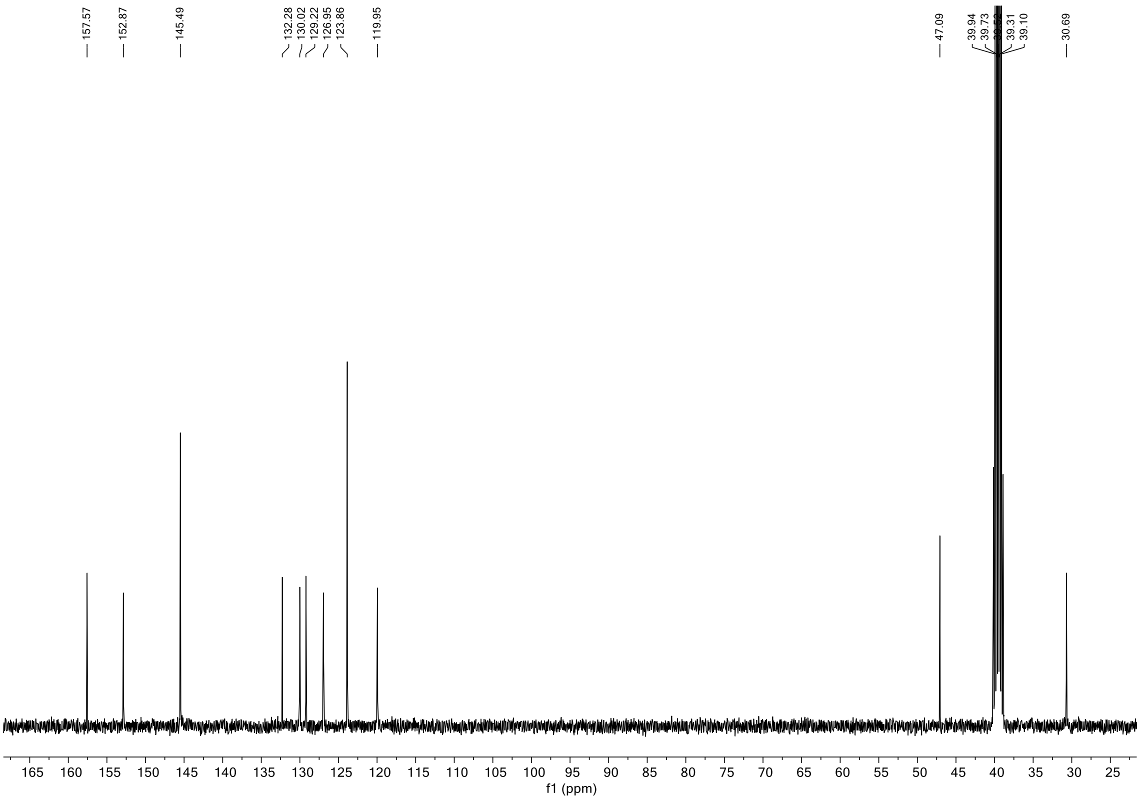

^{13}C NMR spectrum of compound ***cis*-1** (DMSO-d_6, 100 MHz).

3. UV-visible spectroscopy

UV spectra were recorded on a Thermo Scientific BIOMATE 3S UV-vis Visible Spectrophotometer at ambient temperature. Measurements were taken in a 3 mL quartz cuvette with a path length of 10 mm. The UV-visible spectra were recorded between 700 nm and 300 nm and baseline corrected for the buffer used.

4. Photoirradiation experiments

Samples of ***cis*-1** and ***trans*-1** (100 µM in 20 mM potassium phosphate (KPhos) buffer (pH 7.0) and 70 mM KCl) were irradiated with monochromatic light at 405 nm and 520 nm, respectively. The irradiation was performed in a 3 mL quartz cuvette containing a magnetic stirrer and a total volume of 2 mL solution. The irradiation sources were collimated laser diode modules (ThorLabs, CPS405 and CPS520, 4.5 mW, elliptical beam). The photoisomerization was followed by recording the UV-visible spectra at appropriate time points.

For the kinetic experiments, samples of **1** (10 µM) and the appropriate DNA sequence (100 µM by strand) were exposed to ambient room light under otherwise identical conditions. The irradiation was performed in a 3 mL quartz cuvette containing a total volume of 1.5 mL solution. The buffer used was 20 mM potassium phosphate pH 7.0 and 70 mM KCl.

UV–vis and CD experiments involving the trans form were conducted at ambient temperature under continuous light irradiation, with the light source positioned 10 cm from the sample, to suppress thermal back-isomerization.

For photoirradiation experiments conducted in the presence of oligonucleotides and followed by NMR spectroscopy to ensure even irradiation of the sample, the solution (600 μL) was transferred to a 1 mL quartz cuvette containing a magnetic stirrer and irradiated at the appropriate wavelength for the time specified, then transferred back to the NMR tube for analysis.

5. Determination of apparent association or dissociation constants

Apparent association (K_a) or dissociation (K_d) constants for ***cis*-1** and ***trans*-1** were determined through UV-visible spectroscopy titration experiments. The raw spectra were recorded as described in Section 3. The concentration of ligand was fixed at 10 μM in a constant volume of 1.5 mL buffer. The oligonucleotide sequences used were: HIV LTR-III (5′-GGGAGGCGTGGCCTGGGCGGGACTGGGG-3′) and ds26 (5'-CAATCGGATCGAATTCGATCCGATTG-3'). The oligonucleotide was purchased from Eurogentec (Belgium), purified by HPLC and delivered dry. Oligonucleotide concentration was determined by UV-absorbance using a NanoDrop 2000 Spectrophotometer from Thermo Scientific. The buffer used was 20 mM potassium phosphate pH 7.0 and 70 mM KCl or 100 mM pH 7.4 KPhos (ds26). During the titration, aliquots of sample were removed and replaced with aliquots of oligonucleotide to give the required titration points (from a 100 μM stock solution in appropriate buffer containing also 10 μM ligand to maintain constant ligand concentration). NB: the oligonucleotide solution was annealed by heating to 90 °C for 2 minutes and then cooling on ice **prior** to the addition of ligand (to avoid annealing in the presence of ligand). Following addition, the solution was mixed thoroughly and the UV-visible spectrum was acquired immediately. Data were fitted to an independent-and-equivalent-sites binding model (Equation 1) using Prism 7 software, a full derivation of which is provided by (amongst others) Thordarson,[2] adapted to an independent and equivalent sites model by (amongst others) Buurma and Gade.[3] The stoichiometry of the complex (*N*) was chosen as the lowest integer value that provided a satisfactory fit, $R^2 > 0.97$ (N=2, in all cases). The data presented in Figure 3 shows the average values obtained from two independent experiments.

Equation 1:

$$\Delta A = \varepsilon_{\Delta complex}([\text{complex}])$$

$$where:$$

$$[\text{complex}] = \frac{1 + N \cdot K_a \cdot [\text{DNA}]_{tot} + K_a \cdot [\text{ligand}]_{tot} - \sqrt{(1 + N \cdot K_a \cdot [\text{DNA}]_{tot} + K_a \cdot [\text{ligand}]_{tot})^2 - 4 \cdot N \cdot {K_a}^2 \cdot [\text{DNA}]_{tot} \cdot [\text{ligand}]_{tot}}}{2 \cdot K_a}$$

$\Delta A = absorbance\ change\ at\ each\ titration\ point\ relative\ to\ free\ ligand\ (observed\ parameter)$

$\varepsilon_{\Delta complex} =\ change\ in\ the\ molar\ extinction\ coefficient\ between\ free\ ligand\ and\ DNA\ /ligand\ complex\ (fitted\ parameter)$

$N = the\ binding\ stoichiometery\ of\ ligand\ to\ DNA\ (selected\ parameter)$

$K_{\mathrm{a}} = the\ apparent\ association\ constant$ (fitted parameter), $K_{\mathrm{d}} = \frac{1}{K_{\mathrm{a}}}$

$[\mathrm{DNA}]_{tot} = the\ concentration\ of\ added\ DNA\ (known\ parameter)$

$[\mathrm{ligand}]_{tot} = the\ total\ ligand\ concentration\ (fixed\ parameter)$

---

6. Circular dichroism titrations

Circular Dichroism (CD) titrations were recorded using a Jasco J-815 spectrometer fitted with a Peltier temperature controller. Measurements were taken in a quartz cuvette with a path length of 5 mm, at 20 °C, at a 1000 nm/min scanning speed at 1 nm intervals, with a 1 nm bandwidth. The CD spectra were recorded between 600 and 220 nm, and baseline corrected for the buffer used. The oligonucleotide sequences used were: HIV LTR-III (5′-GGGAGGCGTGGCCTGGGCGGGACTGGGG-3′) and ds26 (5'-CAATCGGATCGAATTCGATCCGATTG-3'). The oligonucleotides were purchased from Eurogentec (Belgium), purified by HPLC and delivered dry. Oligonucleotide concentrations were determined by UV-absorbance using a NanoDrop 2000 Spectrophotometer from Thermo Scientific. The oligonucleotide was annealed before use by heating for 2 minutes at 90°C and then placed immediately into ice. The oligonucleotide was at a concentration of 5 µM which gave an OD of 1 and the buffer used was 20 mM potassium phosphate pH 7.0 and 70 mM KCl (HIV TLR-III) or 100 mM pH 7.4 KPhos (ds26). The ligand was added by aliquot from a 1mM stock solution in the appropriate buffer (containing 10% DMSO to ensure solubility). The reported spectrum for each sample represents the average of 3 scans. Data processing was carried out using Prism 7 with an 8-point second order smoothing polynomial applied to all spectra. Observed ellipticities were converted to mean residue ellipticity (θ) = deg cm^2 $dmol^{-1}$ (molar ellipticity).

7. NMR spectroscopy titrations

^{1}H NMR spectra of G-quadruplex sequences were recorded at 278 K* using a 600 MHz Varian VNMRS spectrometer equipped with a triple resonance cryogenically cooled probe head. The oligonucleotide sequences used was HIV LTR-III (5′-GGGAGGCGTGGCCTGGGCGGGACTGGGG-3′). Samples of oligonucleotide were dissolved in 90% H_2O/10% D_2O containing either 20mM potassium phosphate pH 7.0 and 70 mM KCl. All experiments employed sculpted excitation water suppression. The final NMR samples contained 600 µL of 185 µM oligonucleotide. Samples were annealed before use by heating for 2 minutes

at 90°C and then placed immediately into ice. Aliquots of ligand (10 mM in DMSO-d_6) were added to the appropriate yield titration points, the sample was mixed thoroughly and NMR spectra were recorded immediately after the addition of ligand. Data were processed using MestReNova software (version 11.0.2). Resonances were assigned from data provided in the literature by Richter and Phan.[4] Photoirradiation of NMR samples was conducted using the protocol described in Section 4.

* Owing to the fast thermal relaxation of the active *trans*-**1**, it was not possible to study the binding by NMR at ambient temperature, because the ligand reverts to the inactive *cis*-**1** too quickly on the timescale of the NMR experiment. Therefore, we studied the ligand binding at lower temperature (5 °C) in order to discriminate the different binding properties of the two isomers.

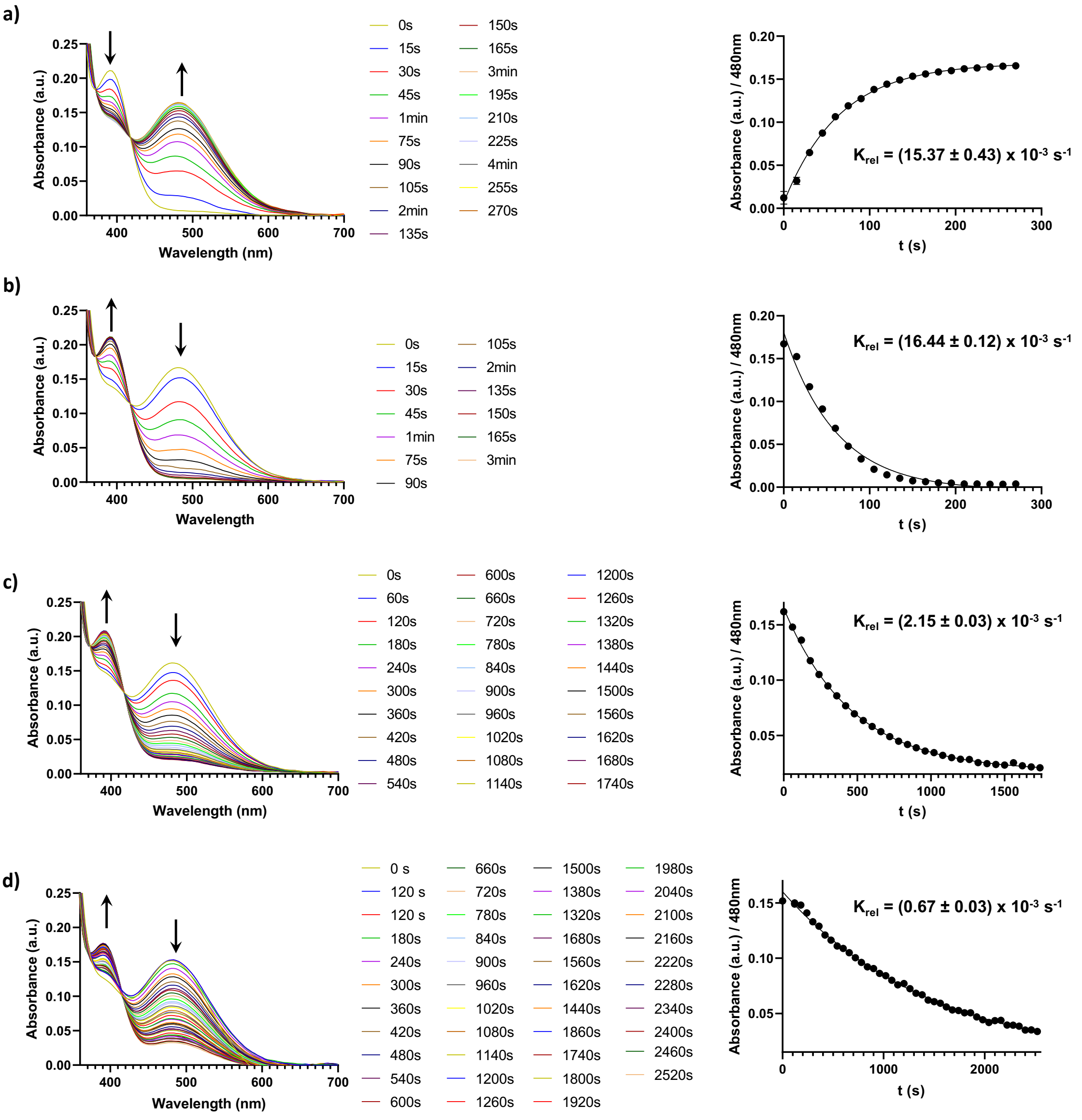

Figure S1. Photoswitching of diazocine *cis*-**1** a) excitation at 405 nm to PSS (*trans*-**1**), b) followed by excitation at 520 nm back to the *cis* diazocine, c) thermal relaxation at 20 °C, and d)

thermal relaxation at 5 ºC at 100 µM of the ligand in 20 mM potassium phosphate buffer (pH 7.0) and 70 mM KCl, to PSS. All constants K_{rel} were determined at 480 nm.

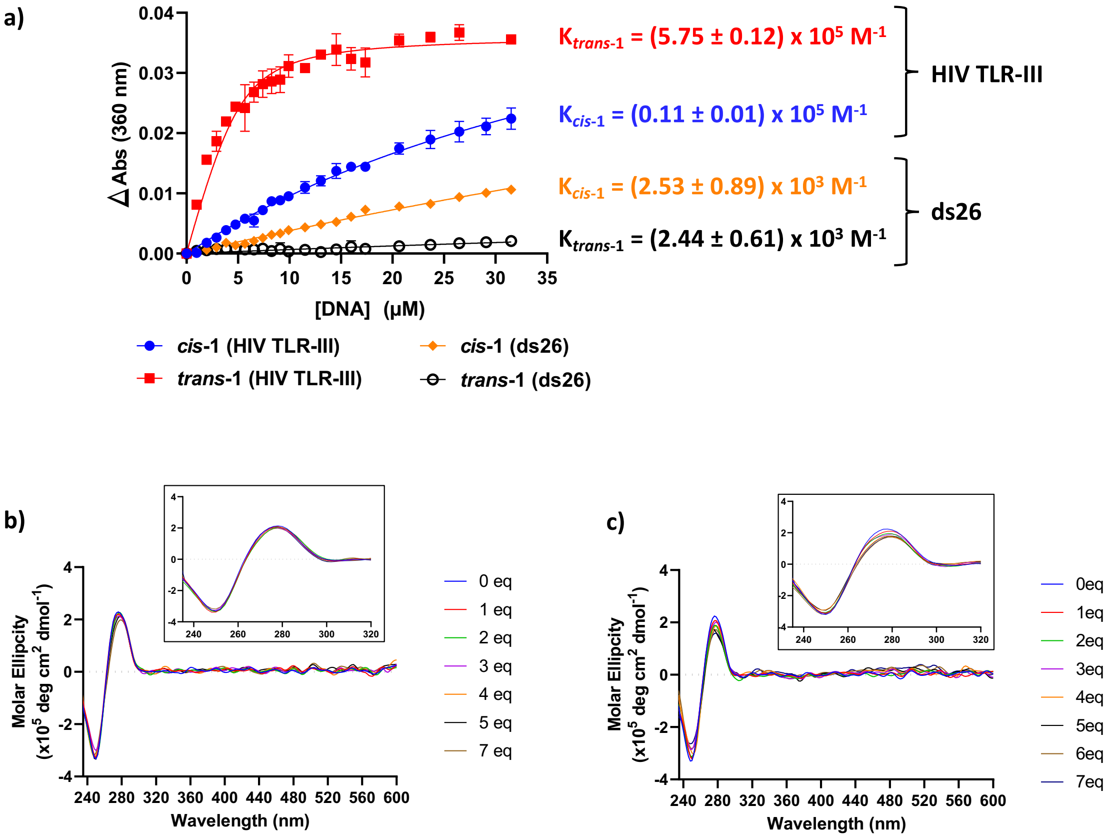

Figure S2. a) UV/visible binding isotherms and association constants of *cis*-**1** and *trans*-**1** to LTR-III and duplex, at room temperature (20 ºC). CD titrations of b) *cis*-**1** and c) *trans*-**1** to duplex ds26, at 20 ºC.

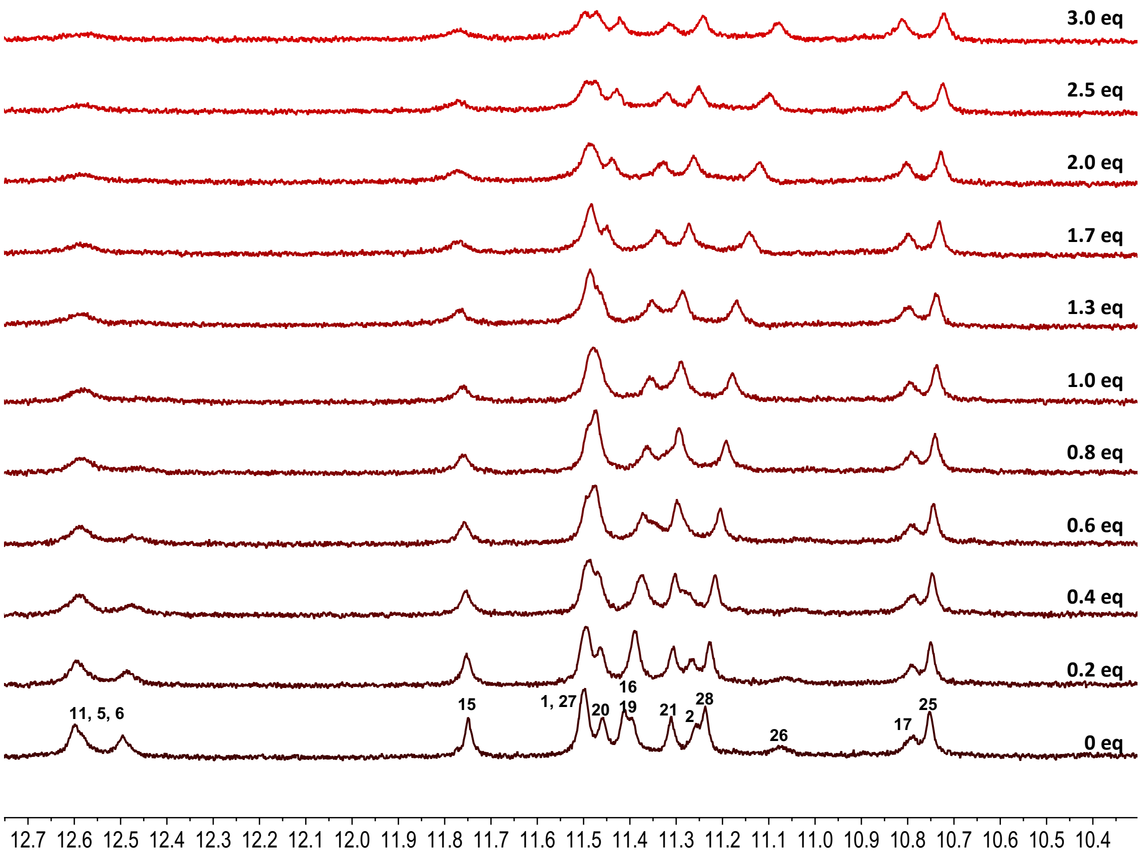

Figure S3a. Stacked NMR spectra (imino region) of LTR-III titrated with ligand *cis*-**1** at 5°C.

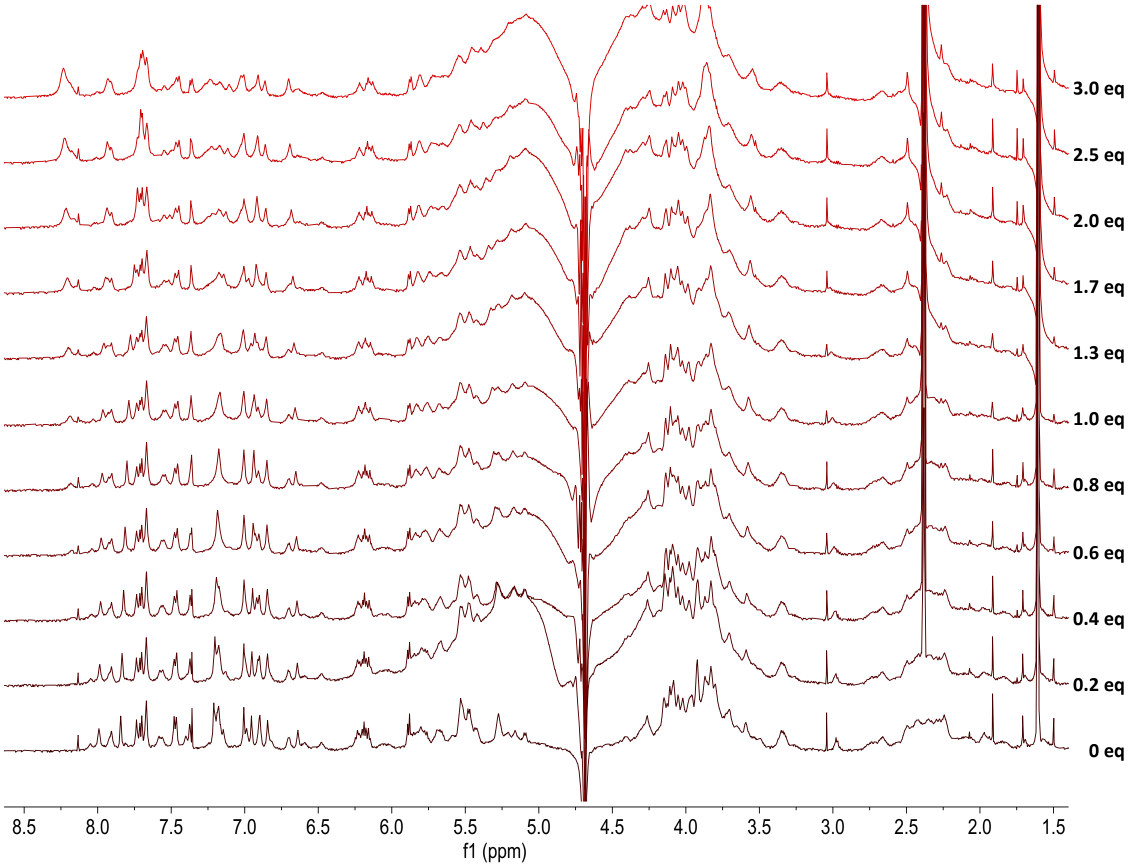

Figure S3b. Stacked NMR spectra (aromatic/carbohydrate/aliphatic region) of LTR-III titrated with ligand *cis*-**1** at 5°C.

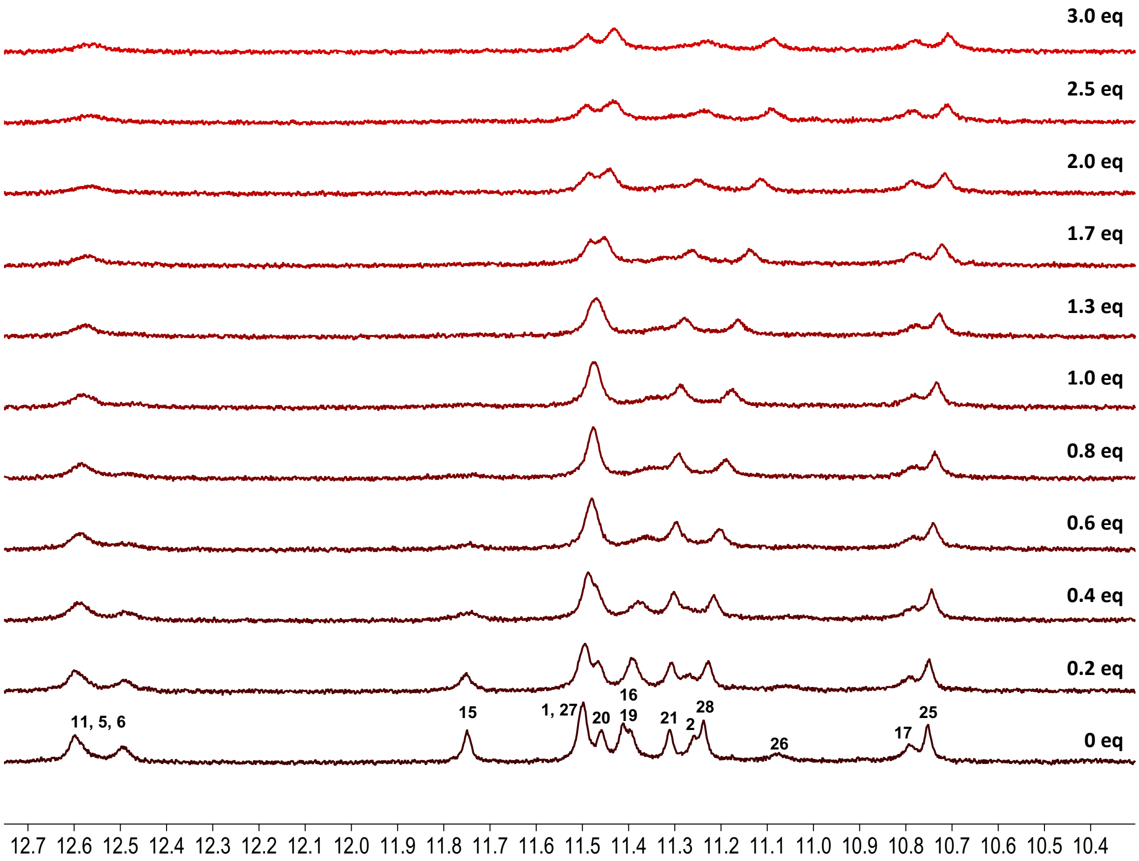

Figure S4a. Stacked NMR spectra (imino region) of LTR-III titrated with ligand *trans*-**1** at 5°C.

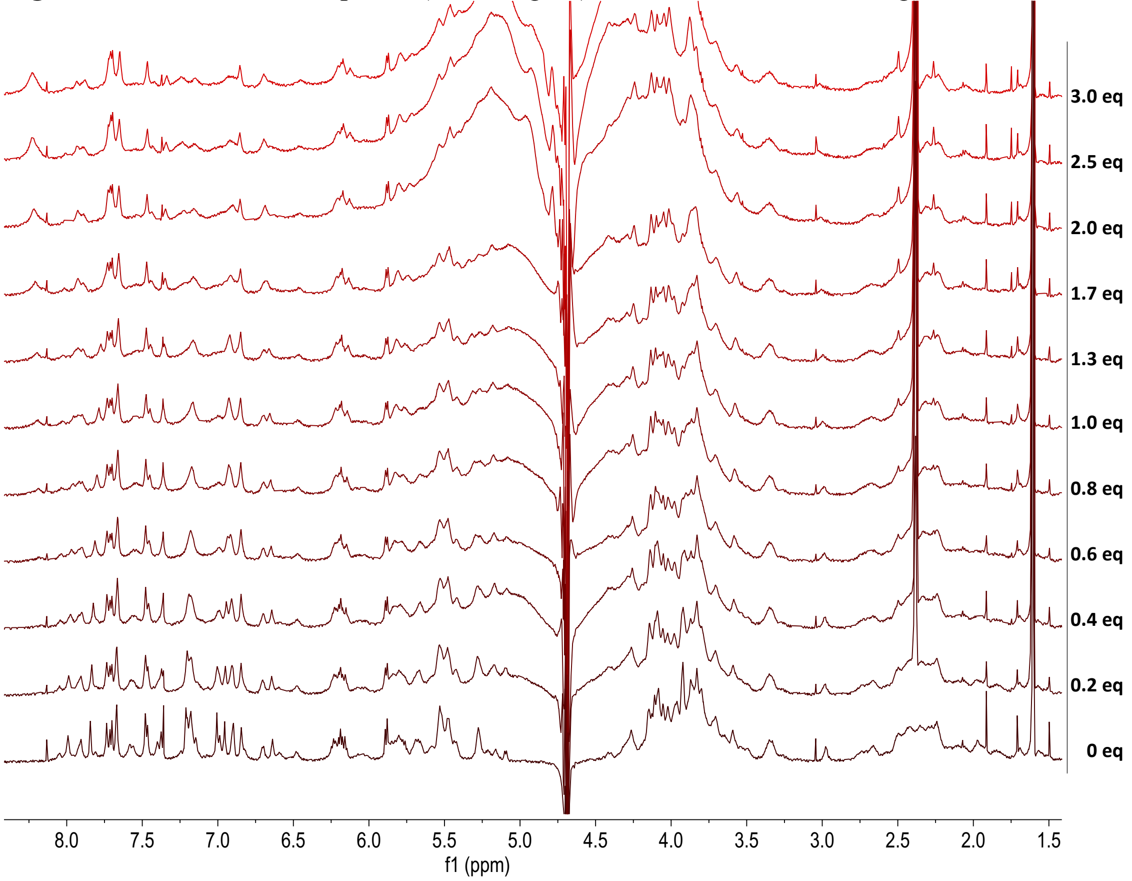

Figure S4b. Stacked NMR spectra (aromatic/carbohydrate/aliphatic region) of LTR-III titrated with ligand *trans*-**1** at 5°C.

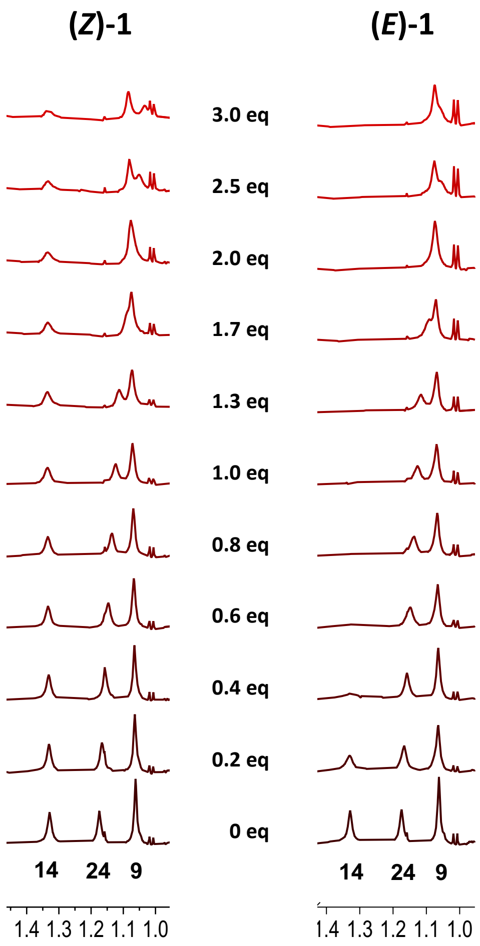

Figures S5. H-NMR of LTR-III thyamine methyl region. NMR titration with *trans*-**1** (right) and *cis*-**1** (left) at 5ºC. Thyamines 14, 24 and 9 highlighted.

8. Molecular dynamics simulations

*8.1 Models for the complexes between the G-quadruplex form of HIV-1 LTR and the cis-**1** and trans-**1** ligands.*

Molecular docking was used to build the complexes between the G-quadruplex form of HIV-1 LTR and the *cis*-**1** and *trans*-**1** ligands. For this, the HIV-1 G-quadruplex NMR structures (all ten models in the structure with PDB code 6H1K)[4] were used. The structures of *cis*-**1** and *trans*-**1** were optimised using the Gaussian16W[5] program at the B3LYP-D3(BJ)/def2-TZVP level of theory.[6] The optimised structures were saved in the PDB format. AutoDockTools[7] was then used to prepare the protein and ligands and convert their PDB files into PDBQT ones. The chemical shifts reported in Figures S3-S5 were used to identify the potential binding sites and thus guide the docking process. According to the NMR data, the potential binding sites are located on the quadruplex-duplex junction (A4 and T14) and nearby bases such as G5, G6, C12, C13, and G15. The numbers of grid points in xyz of docking boxes were 14, 34 and 28, respectively and the spacing was 0.1 nm: this ensures that the box covers these key bases. The grid parameters were saved in the gdf format. The genetic algorithm was used for the docking with settings were as 10: this optimises the solution for molecular docking by mimicking the process of natural selection to find the ligand-receptor complex with the lowest energy. A population size to 150 and a maximum iteration count of 25,000,000 steps was used, with all the other parameters set to the default values. All docking calculations were performed using AutoDock Vina (Version 1.2.0).[8] The DNA-ligand complexes were visualised using PyMOL (Version 2.5),[9] with the ten lowest binding energy ones (one per NMR model) being used as starting points for the MD simulations (Figure S6 and Table S1).

8.2 Molecular dynamics (MD) simulations

ACPYPE[10] was used to obtain the parameters for the *cis*-**1** and *trans*-**1** ligands, using GAFF forcefield[11] and AM1-BCC charge.[12] For *cis*-**1**, restraints were added to the azobenzene part of the ligand, namely to the C3-N2=N4-C15 and C1-C9-C10-C14 dihedrals with a force constant of 1000 kJ $mol^{-1}nm^{-2}$. A third system containing the DNA without any ligands was also prepared. The AMBER14sb_parmbsc1[13] force field was used to describe the nucleic acids, and TIP3P water model[14] was employed. The DNA-ligand complexes were solvated using a cubic box, with a 1 nm solvent layer between the box edges and the solute surface. Two K^+ ions were placed in the center of the three G-quadruplex planes as described in.[15] The systems were neutralised, and a 100 mM was added using K^+ and Cl^- ions. The LINCS[16] algorithm was used to constrain all bonds involving hydrogen atoms within the DNA and ligands, whereas the SHAKE algorithm was used for the water molecules. The integration step was 1.0 fs. Long-range electrostatic interactions beyond 1.2 nm were calculated using the smooth particle mesh ewald (SPME) method.[17] Van der Waals were cut off at 1.2 nm. Then, a two-step energy minimisation process

was performed: first, 2000 steps of steepest descent followed by 2000 steps of conjugate gradient, with the maximum force set to 100 kJ mol^{-1} nm^{-2}.

Next, all systems were equilibrated at a constant temperature of 300 K and pressure of 1 atm for 500 ps, with all heavy atoms restrained at their starting position using a force constant of 1000 kJ $mol^{-1}nm^{-2}$. All production runs started from these equilibrated conformations. Temperature was maintained at 300 K using a v-rescale[18] thermostat with separate coupling of the DNA-ligand and non-DNA using a time constant of 0.2 ps. Pressure was kept at 1 atm using Parrinello–Rahman[19] barostat, with a time constant of 10 ps. Frames, velocities and energies were saved every 10 ps.

The root-mean-square deviations (RMSD) of the DNA, G-quadruplex and ligands relative to their starting conformation were analysed using MDAnalysis (version 2.7.0).[20] The stability of the simulations was assessed by calculating the time evolution of the root-mean-square deviation (RMSD) profiles for the whole DNA (Figure S7) and G-quadruplex bases (Figure S8). The time evolution of the RMSD for the *trans*-**1** and *cis*-**1** ligands was used to evaluate the stability of the lowest energy binding pose identified from docking (Figure S9). Ligand RMSD is a simple metric to assess the stability of binding poses; it compares the conformations adopted in MD simulations with a reference binding mode (the lowest energy binding modes in each model for two ligands determined by AutoDock Vina).

Binding energies between the DNA and the *cis*-**1** and *trans*-**1** ligands were calculated using the molecular mechanics energies combined with the Poisson‐Boltzmann and surface area continuum solvation (MM-PBSA).[21]

MM-PBSA, which offers improved accuracy over molecular docking for estimating binding affinities, is widely used in the biomolecular modelling field for its balance between computational efficiency and qualitative insight.[21] Given this, this approach was selected for determining binding energy differences between *cis*-**1** and *trans*-**1** ligands as it offers an effective balance between computational cost and the qualitative insights necessary to distinguish key binding features across the two systems. For a comprehensive overview of the MM-PBSA methodology, including its limitations and comparisons with alternative approaches for estimating binding energies, readers are encouraged to consult the following recent reviews.[21]

Binding energies for individual models were calculated using gmx_MMPBSA (version 1.6.3) tool,[22] based on 10000 conformations per model (one frame every 20 ps). To obtain average binding energies, conformational data from all ten individual models were combined, totaling 200020 frames per ligand-G4 complex.

To compare binding energies of the two ligand isomers (cis vs. trans), two complementary statistical approaches were employed, each addressing different aspects of our data sets. First, within each DNA structural model, paired t-tests were performed frame-by-frame, for the cis and trans binding energies

within the same frames which are inherently paired and correlated. This approach directly quantifies ligand differences under identical structural conditions.
However, because our dataset comprises multiple structural models (10 independent NMR structures), variation between these models may significantly affect overall binding energies. To account for this inter-model variability, a linear mixed-effects model (LMM) was utilized, incorporating ligand identity as a fixed effect and structural model as a random effect. This allowed us to robustly estimate the ligand-specific effects while correcting for systematic differences between individual models. Compared to a simple pooled analysis or model-averaged t-test, the mixed-effects modeling approach provides more accurate estimates of ligand effects by appropriately partitioning and controlling structural variability.[23]
Principal component analysis (PCA)[24] was used to determine the relationship between the 3D conformational features of the binding sites and interactions between ligand and DNA bases for all twenty trajectories. The C1' atom (the carbon atom linking the deoxyribose and base in the DNA), together with all heavy atoms from the ligand, were used for the PCA.
Density-based spatial clustering of applications with noise (DBSCAN)[25] was used for clustering the trajectories with 1epsilon (eps) and 50 min-samples for each ligand and extracting the centroid frame. The centre of mass (i.e. the average position in PCA space) of all frames in each cluster was calculated, and the frame with the closest distance to the centre of mass (centroid frame) was selected as the keyframe, which can best represent the characteristics of the cluster.

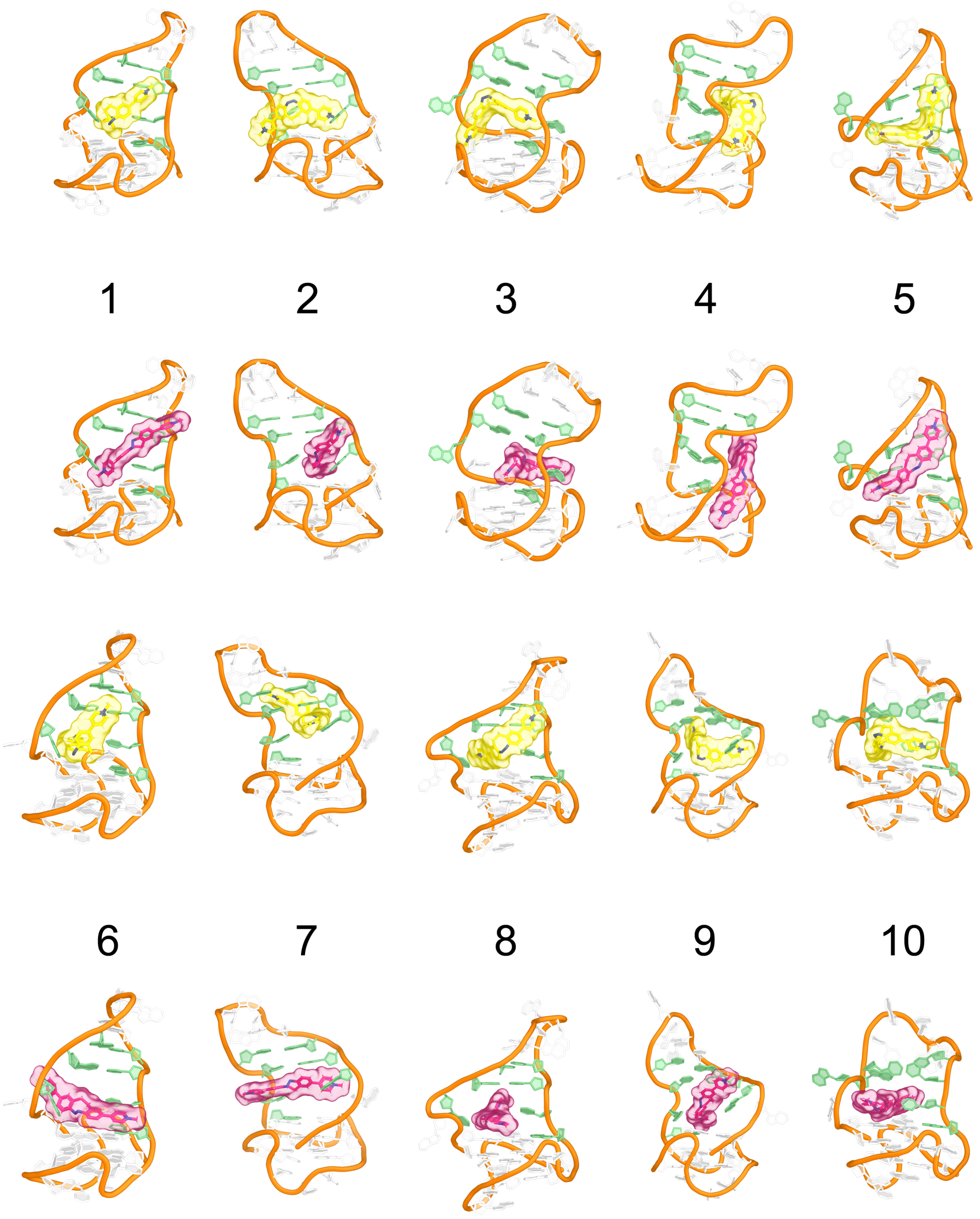

Figure S6. *trans*-**1** and *cis*-**1** binding modes for each of the ten models in PDB 6H1K as predicted by AutoDock Vina.

As can be seen in Figure S8, the G-quadruplex region remains generally stable in all systems, except for the model2:*trans*-**1** complex. In the model2:*cis*-**1** complex, G28 underwent a structural change and moved away from G17, G21, and G25, thus disrupting the Hoogsteen base pairs, causing the increase in RMSD observed in Figure S7. The RMSD profiles for the whole DNA segment are higher than the G-quadruplex region, highlighting the highly flexible nature of the duplex region (Figure S7).

As shown in Figure S9, the RMSD for all ten models indicates that, in general the *trans*-**1** and *cis*-**1** conformations remain relatively close to their initial binding poses. A diversity of behaviors can be observed for the different *trans*-**1** and *cis*-**1** complexes during the simulations. In all simulated complexes, the ligands remained in direct contact with the DNA, with no unbinding observed during the simulation time.

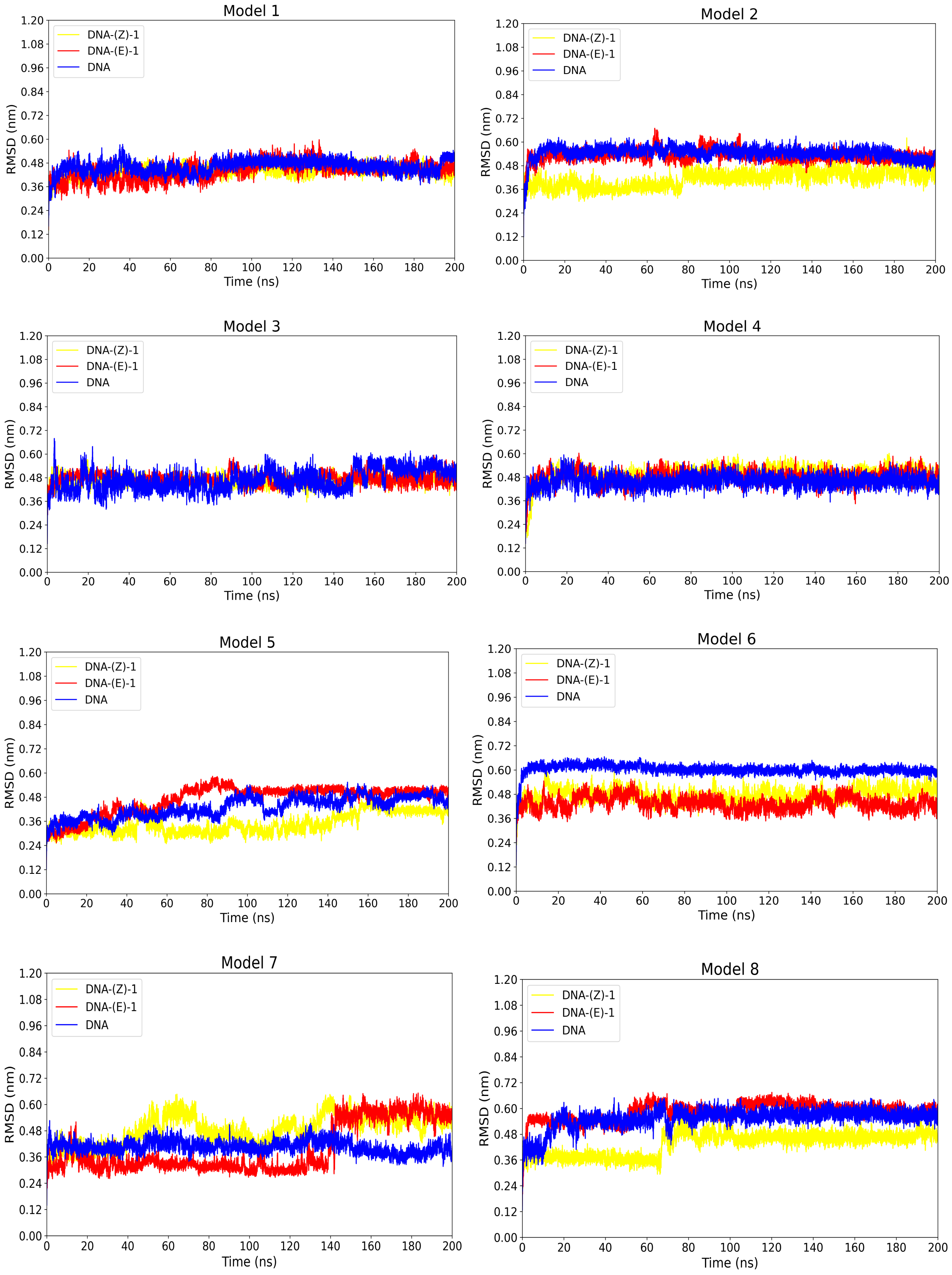

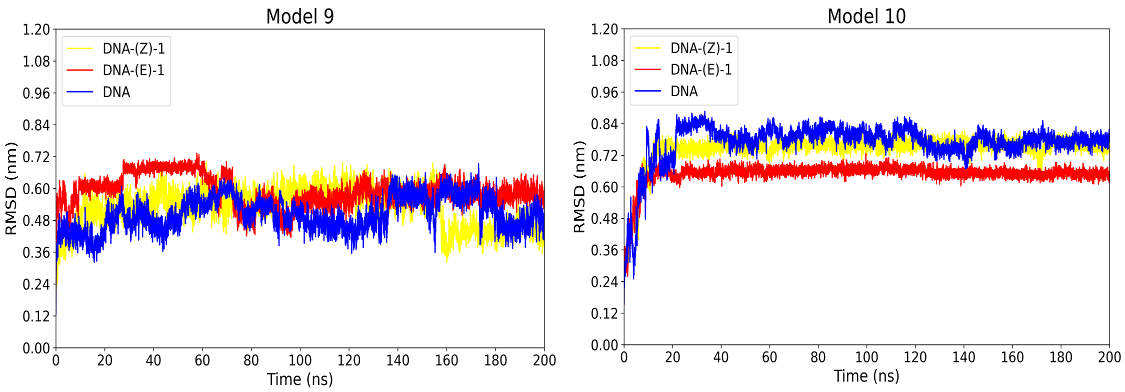

Figure S7. Time evolution of the RMSD for the DNA bases relative to its starting conformations. The yellow lines represent the DNA:*cis*-**1** complexes, the red line the DNA:*trans*-**1** ones, and the blue lines represent the DNA system without ligand bound.

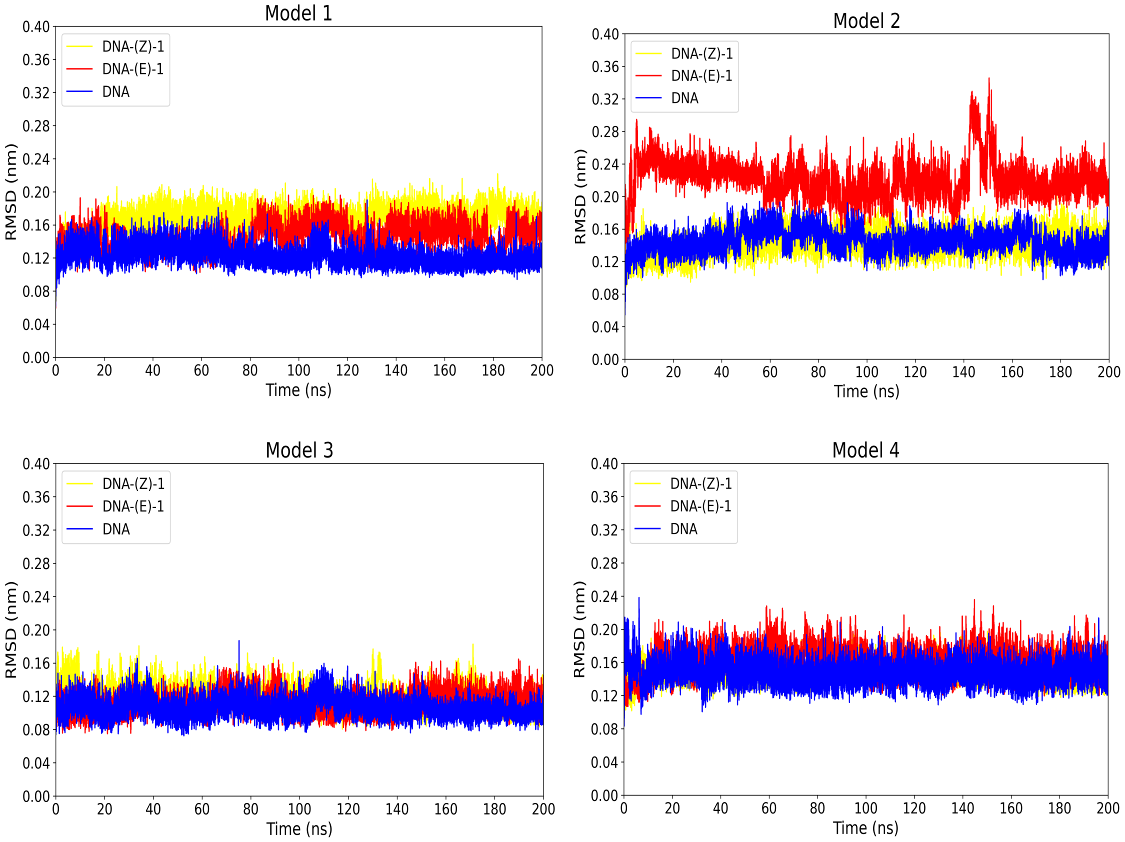

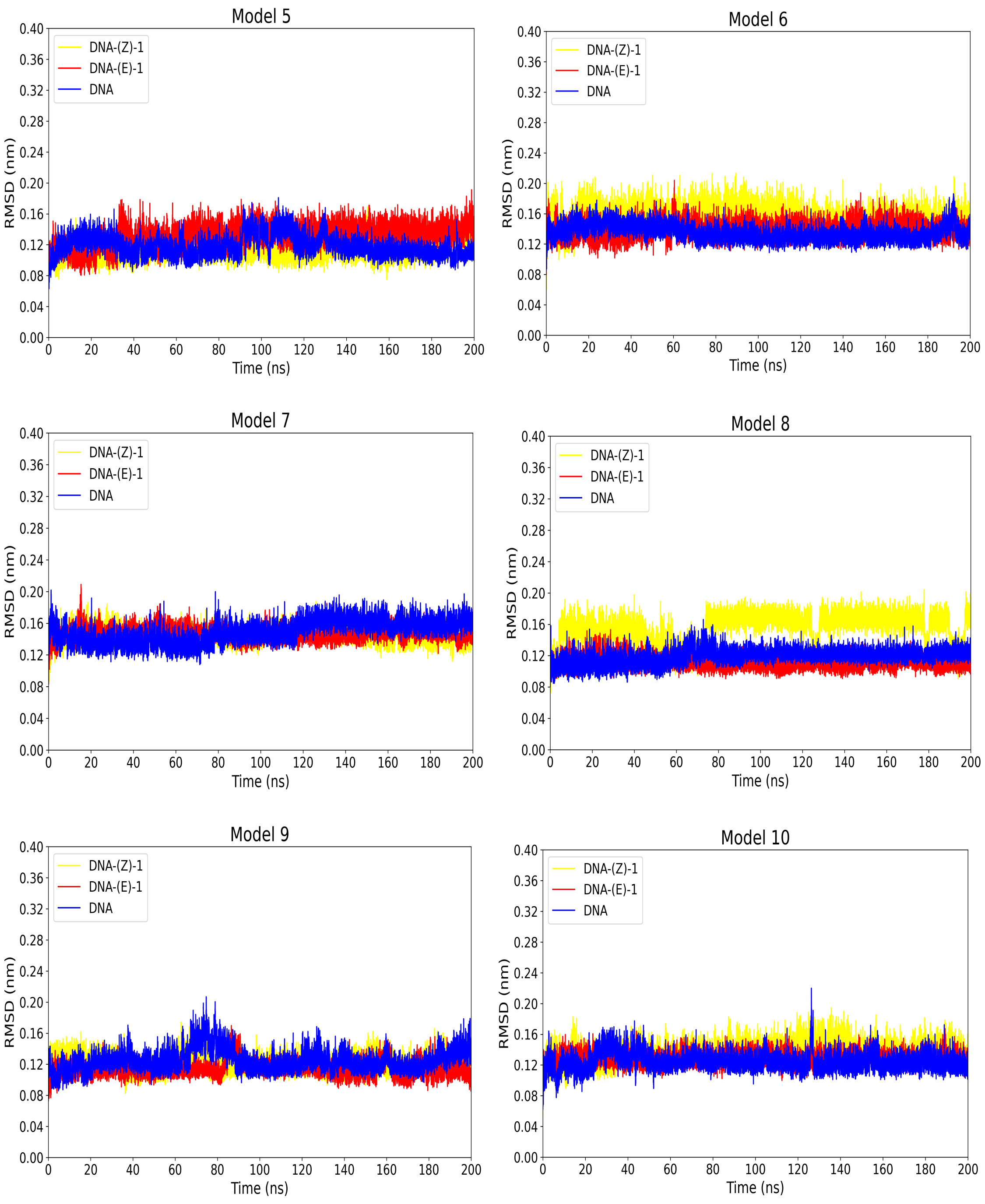

Figure S8. Time evolution of the RMSD of G-quadruplex bases relative to its starting conformations. The yellow lines represent the DNA:*cis*-**1** complexes, the red lines the DNA:*trans*-**1** ones, and the blue lines represent the DNA system without ligand bound.

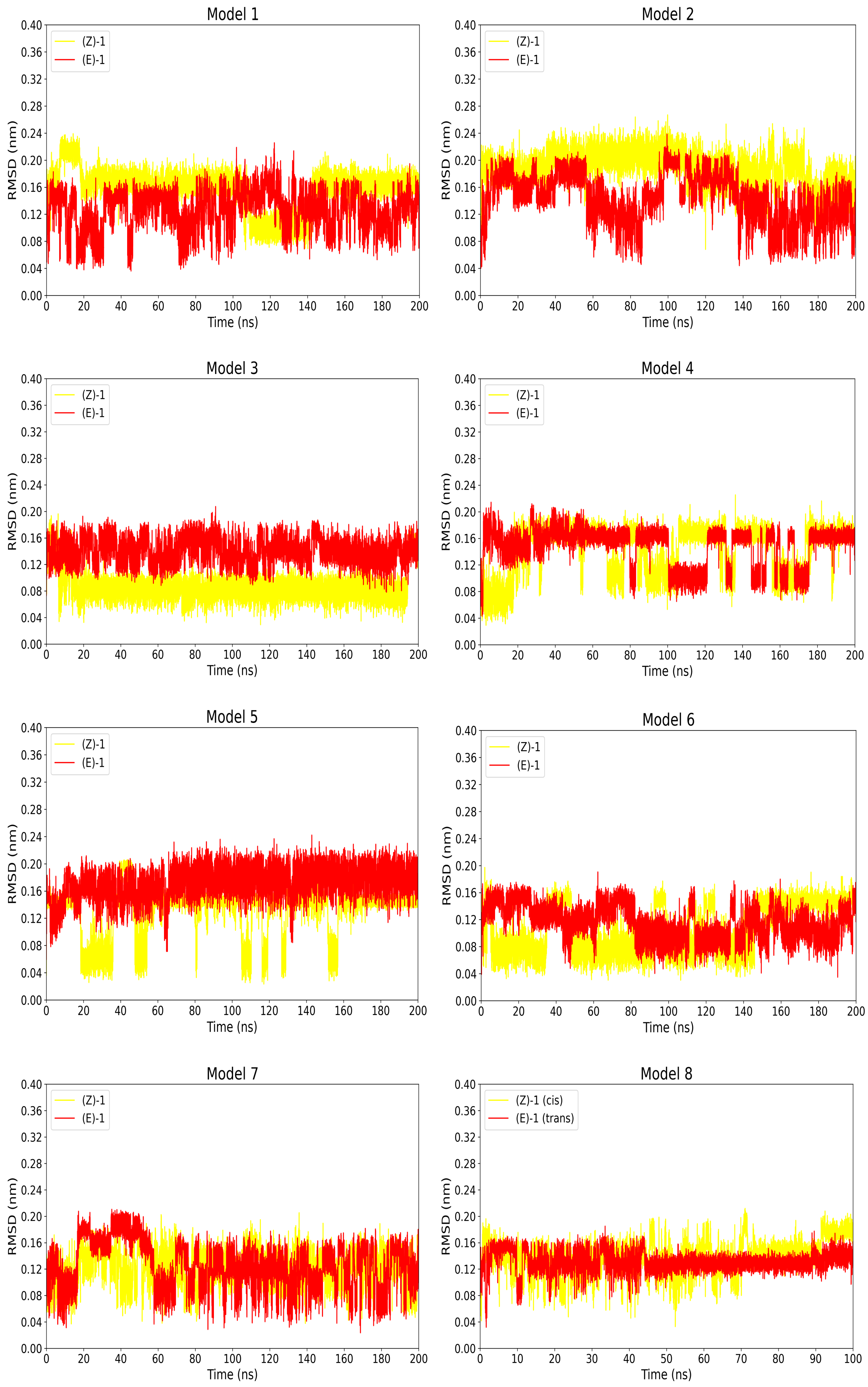
Model 1
(Z)-1
(E)-1
RMSD (nm)
Time (ns)
Model 2
(Z)-1
(E)-1
RMSD (nm)
Time (ns)
Model 3
(Z)-1
(E)-1
RMSD (nm)
Time (ns)
Model 4
(Z)-1
(E)-1
RMSD (nm)
Time (ns)
Model 5
(Z)-1
(E)-1
RMSD (nm)
Time (ns)
Model 6
(Z)-1
(E)-1
RMSD (nm)
Time (ns)
Model 7
(Z)-1
(E)-1
RMSD (nm)
Time (ns)
Model 8
(Z)-1 (cis)
(E)-1 (trans)
RMSD (nm)
Time (ns)

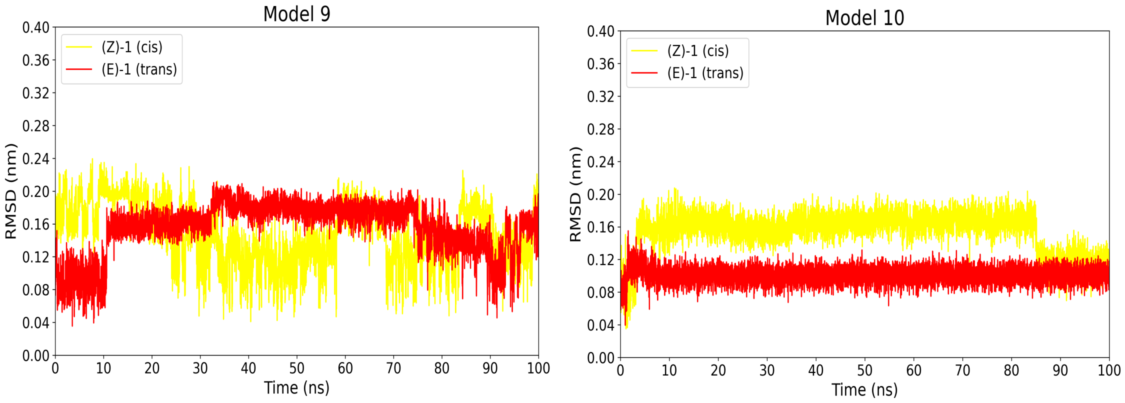

Figure S9. Time evolution of the RMSD of the ligands relative to their starting binding modes. The yellow and red lines represent the *cis*-**1** and *trans*-**1** ligands, respectively.

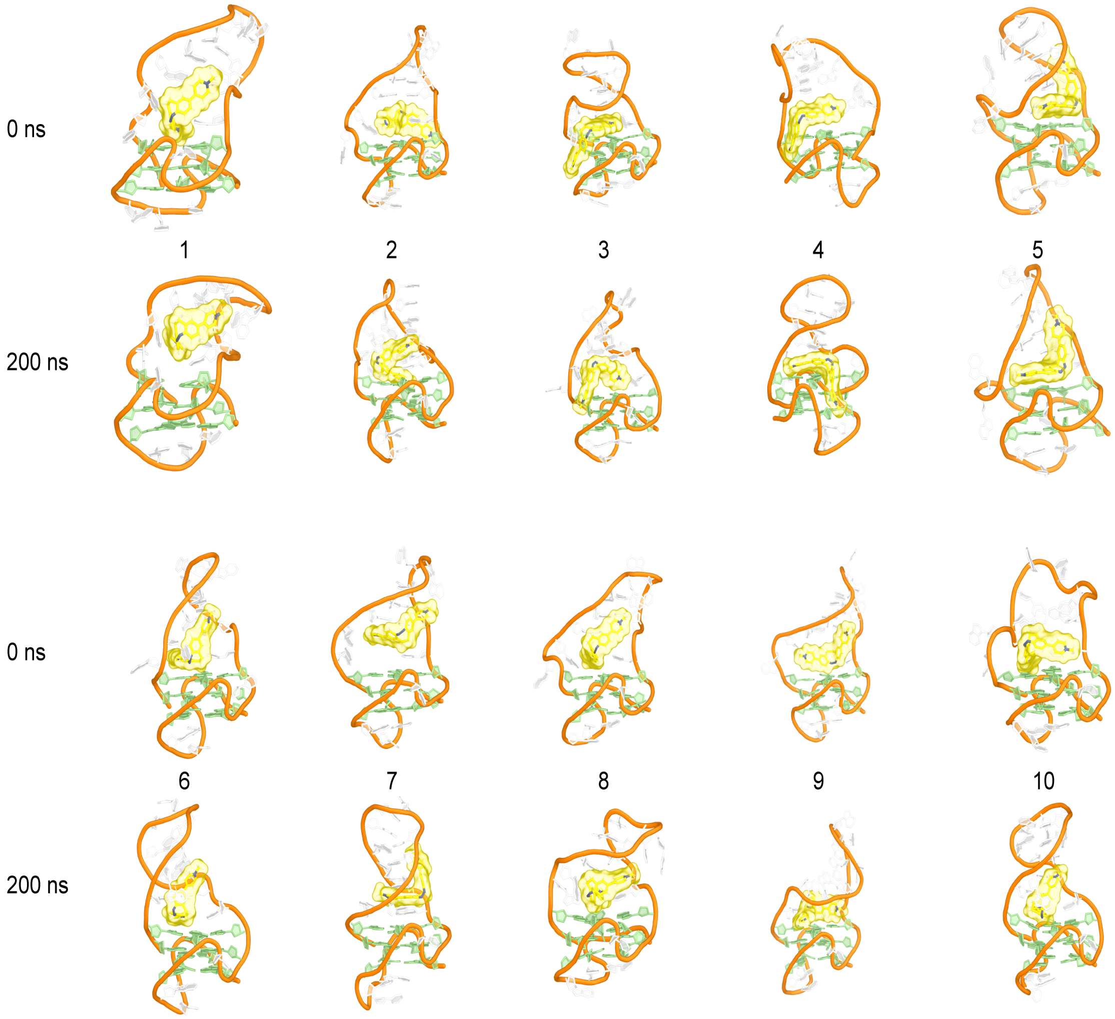

Figure S10. Starting (t=0 ns) and final (t=200 ns) conformations for the DNA:*cis*-**1** complexes. The green sticks identify the G-quadruplex planes. The *cis*-**1** ligand is shown with yellow spheres. Please zoom in on the image for detailed visualization.

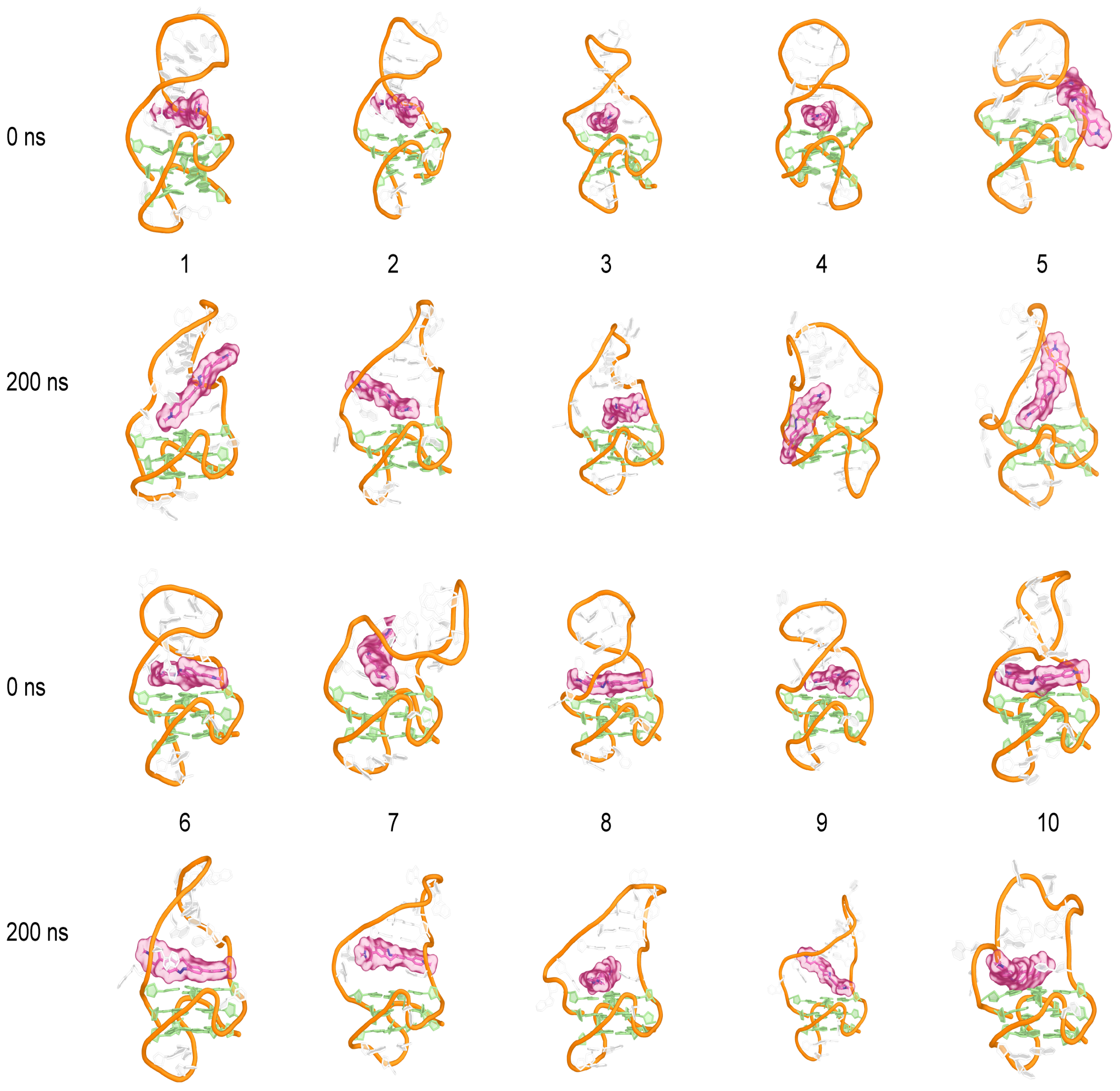

Figure S11. Starting (t=0 ns) and final (t=200 ns) conformations for the DNA:*trans*-**1** complexes. The green sticks identify the G-quadruplex planes. The *trans*-**1** ligand is shown with red spheres. Please zoom in on the image for detailed visualization.

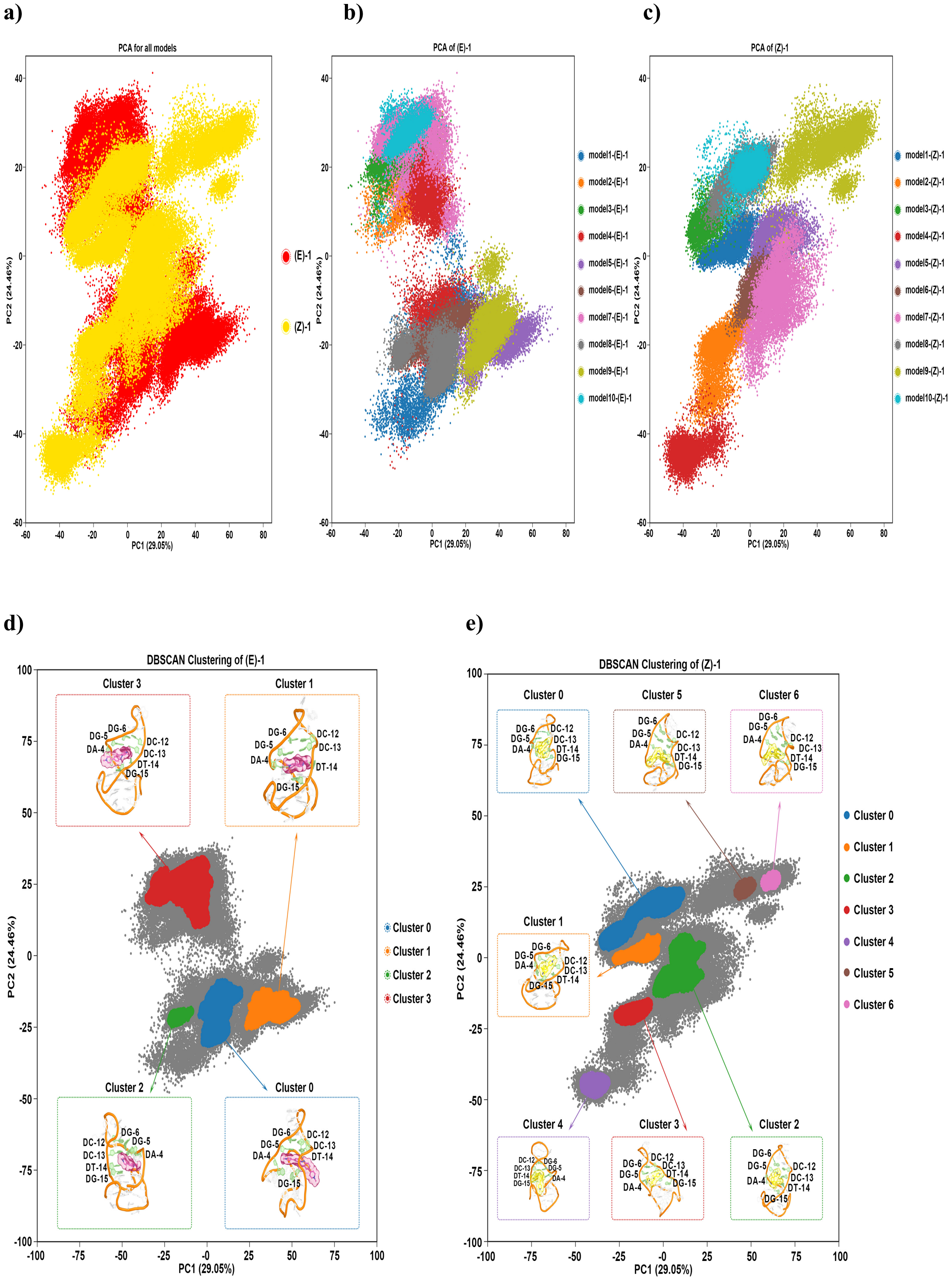

Figure S12. a) Principal component analysis (PCA) and clustering analysis for the DNA:*trans*-**1** and DNA:*cis*-**1** simulations. PCA for the pocket (using the C1' carbon of A4, G5, G6, 12C,

13C, T14 and G15) over the 20 trajectories simulated. The yellow dots represent the *cis*-**1** ligand, whereas the red ones show *trans*-**1**. **b**) PCA for the pocket for the ten models of the DNA:*trans*-**1** complex. **c**) PCA for the pocket for the ten models of the DNA:*cis*-**1** complex. **d**) DBscan clustering for DNA:*trans*-**1** complex and the centroid frames of each cluster. **e**) DBscan clustering for DNA:*cis*-**1** complex and the centroid frames of each cluster. Please note that models 1, 3, 5 and 9 for the DNA:*trans*-**1** complex and models 4 and 9 for the DNA:*cis*-**1** complex sample unique regions of the space showing no overlap between the conformations adopted by the pockets and significant differences in the interactions between the ligands and the DNA.

Table S1. Binding energy for the complexes shown in Figure S6. The binding energies were calculated using Autodock Vina.

models	*trans*-**1** Binding energy (kcal/mol)	*cis*-**1** Binding energy (kcal/mol)
1	-8.1	-8.8
2	-8.7	-9.1
3	-9.1	-8.9
4	-8.8	-8.8
5	-9	-8.4
6	-8.7	-9.1
7	-7.2	-7.4
8	-9.3	-8.9
9	-8.2	-8.8
10	-9.7	-9.2

Table S2. MM-PBSA binding energies for the *cis*-**1** and *trans*-**1** complexes. A Student's t-test was used to compare the binding energies for the *cis*-**1** and *trans*-**1** complexes and assess the statistical significance of the results. For this test, the sample size was 10000, and it was assumed that the two samples were independent.

models	*cis*-**1** Binding energy (kcal/mol)	*trans*-**1** Binding energy (kcal/mol)	T-statistic	*p* value
1	-48.09	-43.54	-87.19	0.00
2	-48.49	-46.58	-33.37	0.00
3	-45.02	-46.54	18.91	0.00
4	-35.40	-41.48	111.86	0.00
5	-29.50	-35.11	69.69	0.00
6	-42.77	-37.70	-76.28	0.00

7	-43.61	-46.34	52.60	0.00
8	-39.26	-40.86	20.89	0.00
9	-41.23	-46.63	67.70	0.00
10	-44.33	-40.94	-57.02	0.00
Average	-41.77	-42.57		

Table S3. Statistical analysis of average MM-PBSA binding energies for the *cis*-**1** and *trans*-**1** ligands. A linear mixed-effects model[25] was used to assess the significance of the average energy difference reported in Table S2. The average binding energies were determined using the data from all ten ligand-G4 complexes. Ligand identity was included as a fixed effect and model ID as a random intercept, with restricted maximum likelihood estimation (REML). The intercept represents the average binding energy for *cis*-**1** (-41.771 kcal/mol), while *trans*-**1** showed a statistically significant energy decrease of -0.802 kcal/mol relative to *cis*-**1** ($p < 0.001$).

Effect	Coefficient	Std. Error	z-value	p-value
Intercept (*cis*-**1**)	-41.771	1.552	-26.914	< 0.001
Ligand (*trans*-**1** vs *cis*-**1**)	-0.802	0.023	-34.352	< 0.001

9. References

[1] M. S. Maier, K. Hull, M. Reynders, B. S. Matsuura, P. Leippe, T. Ko, L. Schaffer, D. Trauner, *J Am Chem Soc* **2019**, *141*, 17295-17304.

[2] D. Brynn Hibbert, P. Thordarson, *Chem Commun (Camb)* **2016**, *52*, 12792-12805.

[3] L. Hahn, N. J. Buurma, L. H. Gade, *Chemistry (Weinheim an der Bergstrasse, Germany)* **2016**, *22*, 6314-6322.

[4] E. Butovskaya, B. Heddi, B. Bakalar, S. N. Richter, A. T. Phan, *J Am Chem Soc* **2018**, *140*, 13654-13662.

[5] Frisch M J, Trucks G W, Schlegel H B, et al. Gaussian 16, Revision A. 03, Gaussian[J]. Inc., Wallingford CT, 2016, 3.

[6] aT. Yanai, D. P. Tew, N. C. Handy, *Chemical Physics Letters* **2004**, *393*, 51-57; bF. Weigend, *Physical Chemistry Chemical Physics* **2006**, *8*, 1057-1065.

[7] Huey R, Morris G M, Forli S. Using AutoDock 4 and AutoDock vina with AutoDockTools: a tutorial[J]. The Scripps Research Institute Molecular Graphics Laboratory, 2012, 10550(92037): 1000.

[8] O. Trott, A. J. Olson, *Journal of Computational Chemistry* **2010**, *31*, 455-461.

[9] DeLano W L. Pymol: An open-source molecular graphics tool[J]. CCP4 Newsl. Protein Crystallogr, 2002, 40(1): 82-92.

[10] Sousa da Silva A W, Vranken W F. ACPYPE-Antechamber python parser interface[J]. BMC research notes, 2012, 5: 1-8.

[11] J. Wang, R. M. Wolf, J. W. Caldwell, P. A. Kollman, D. A. Case, *Journal of Computational Chemistry* **2004**, *25*, 1157-1174.

[12] A. Jakalian, D. B. Jack, C. I. Bayly, *J Comput Chem* **2002**, *23*, 1623-1641.

[13] aJ. A. Maier, C. Martinez, K. Kasavajhala, L. Wickstrom, K. E. Hauser, C. Simmerling, *J Chem Theory Comput* **2015**, *11*, 3696-3713; bI. Ivani, P. D. Dans, A. Noy, A. Pérez, I. Faustino, A. Hospital, J. Walther, P. Andrio, R. Goñi, A. Balaceanu, G. Portella, F. Battistini, J. L. Gelpí, C. González, M. Vendruscolo, C. A. Laughton, S. A. Harris, D. A. Case, M. Orozco, *Nature Methods* **2016**, *13*, 55-58.

[14] P. Mark, L. Nilsson, *The Journal of Physical Chemistry A* **2001**, *105*, 9954-9960.

[15] M. Castelli, F. Doria, M. Freccero, G. Colombo, E. Moroni, *Journal of Chemical Theory and Computation* **2022**, *18*, 4515-4528.

[16] B. Hess, H. Bekker, H. J. C. Berendsen, J. G. E. M. Fraaije, *Journal of Computational Chemistry* **1997**, *18*, 1463-1472.

[17] U. Essmann, L. Perera, M. L. Berkowitz, T. Darden, H. Lee, L. G. Pedersen, *The Journal of Chemical Physics* **1995**, *103*, 8577-8593.

[18] G. Bussi, D. Donadio, M. Parrinello, *The Journal of Chemical Physics* **2007**, *126*.

[19] R. Martoňák, A. Laio, M. Parrinello, *Physical Review Letters* **2003**, *90*, 075503.

[20] N. Michaud-Agrawal, E. J. Denning, T. B. Woolf, O. Beckstein, *Journal of Computational Chemistry* **2011**, *32*, 2319-2327.

[21] aS. Genheden, U. Ryde, *Expert Opin Drug Discov* **2015**, *10*, 449-461; bC. Wang, P. H. Nguyen, K. Pham, D. Huynh, T. B. Le, H. Wang, P. Ren, R. Luo, *J Comput Chem* **2016**, *37*, 2436-2446.

[21] Genheden S, Ryde U. The MM/PBSA and MM/GBSA methods to estimate ligand-binding affinities[J]. Expert opinion on drug discovery, 2015, 10(5): 449-461.

[22] Valdés-Tresanco M S, Valdés-Tresanco M E, Valiente P A, et al. gmx_MMPBSA: a new tool to perform end-state free energy calculations with GROMACS[J]. *J Chem Theory Comp*, 2021, 17(10): 6281-6291.

[23] Pinheiro J, Bates D. Mixed-effects models in S and S-PLUS[M]. Springer science & business media, 2000.

[24] Abdi H, Williams L J. Principal component analysis[J]. Wiley interdisciplinary reviews: computational statistics, 2010, 2(4): 433-459.

[25] Khan K, Rehman S U, Aziz K, et al. DBSCAN: Past, present and future[C]//The fifth international conference on the applications of digital information and web technologies (ICADIWT 2014). IEEE, 2014: 232-238.
